# How do theme park attributes influence visitors’ positive emotions? Nonlinear effects and spatial heterogeneity in Shanghai’s theme parks

**DOI:** 10.1371/journal.pone.0344051

**Published:** 2026-06-18

**Authors:** Xuehui Guo, Xue Li, Guishuo Ren, Mingze Gao, Chunli Han

**Affiliations:** 1 College of Physical Education and Sport Science, Qufu Normal University, QuFu, China; 2 School of Medical Rehabilitation, QuFu Yuan Dong Vocational and Technical College, QuFu, China; 3 Development Planning Division, Shandong Sport University, JiNan, China; Zhejiang Agriculture and Forestry University: Zhejiang A and F University, CHINA

## Abstract

Against the backdrop of sustained growth in experience-oriented urban leisure consumption, theme parks serve as key venues for visitor experiences. A clearer account is needed of how park attributes and urban spatial structures jointly relate to visitors’ positive emotions. This study examines 106 theme parks in Shanghai to quantify visitors’ positive emotions and identify the supply-side attributes associated with them, while also examining park clustering patterns at the urban spatial scale and their relationship with emotional variations. The research integrates online reviews, Points of Interest (POI), and geospatial data to conduct emotional measurement, spatial pattern analysis, interpretive predictive modeling, and supplementary regression tests. Results indicate that park distribution exhibits significant clustering, yet emotional levels within hotspots are inconsistent. IP strength, service quality, and green-space leisure are the attributes most strongly associated with visitors’ positive emotions. Among these, only IP strength showed robust statistical evidence of nonlinearity in the supplementary spline and segmented regressions; service quality showed a possible inflection trend but was not statistically significant; green-space leisure generally exhibited a stable positive association. The study concludes that emotional experiences in theme parks are influenced not only by perceptible cues within the park but also shaped by the structure of urban spatial supply. Therefore, at the operational level, priority should be given to enhancing cross-touchpoint thematic consistency, process predictability, and the provision of decentralized restorative nodes; at the urban level, efforts should focus on optimizing connectivity to hotspot areas, mitigating the externalities of crowding, and promoting the decentralization of recreational supply.

## 1. Introduction

Against the backdrop of sustained growth in experience-oriented leisure and tourism consumption, theme parks, as significant recreational spaces, have garnered extensive academic attention in the tourism sector [1]. Existing research indicates that visitors’ positive emotions in theme parks not only relate to satisfaction [2] and behavioral intentions [3], but also serve as a key indicator of experience quality [4]. However, for theme parks in megacities, positive emotions cannot be understood solely as the direct outcome of in-park attractions, thematic narratives, or service processes. Theme parks are embedded within a supply structure comprising urban transportation, spatial agglomeration, and surrounding recreational resources. Differences in location shape visitors’ access conditions, surrounding environments, and agglomeration pressures associated with different locations collectively alter visitors’ visitation conditions, comparison benchmarks, and experience expectations [5,6]. Therefore, any discussion of how theme park attributes influence visitors’ positive emotions cannot be separated from the urban spatial scale.

Shanghai provides a suitable case study for examining these issues. On the one hand, Shanghai hosts a diverse array of theme parks, including international brands and local attractions, giving the city both a large market and broad sample coverage [7,8]. On the other hand, these parks are not uniformly distributed but are embedded within distinct spatial supply patterns, exhibiting significant differences in accessibility, prime locations, surrounding recreational environments, and levels of agglomeration [5]. Against this backdrop, even parks of similar types may exhibit variations in visitors’ positive emotions due to differing urban spatial conditions. Accordingly, Shanghai provides a suitable case through which differences in park attributes and urban spatial contexts can be integrated within a single analytical framework.

Although existing research has established a relatively robust body of evidence regarding emotional experiences [9] and satisfaction [10], and as methodologies have evolved, researchers have begun to attempt to visualize theme park visitors’ emotions by combining social media analysis with geospatial analysis [11,12], providing new pathways for understanding emotional differences based on real-world contextual data. However, three shortcomings remain. First, existing research has primarily focused on individual parks or single scenarios, and there remains a lack of a comparative measurement framework for positive emotional differences among multiple parks at the urban scale [11], making it difficult to explain why parks of the same type exhibit different emotional outcomes in different locations. Second, regarding attribute characterization, many studies still rely primarily on questionnaires or limited subjective indicators, with few incorporating multidimensional attributes—such as IP strength, service quality, green-space leisure, and spatial organization—within the same framework to compare their relative contributions [13–15]. Third, although social media, spatial data, and interpretable machine learning provide new tools for research [6], spatial analysis risks remaining merely a demonstration of methodology rather than genuinely contributing to the explanation of mechanisms underlying emotional differences if it lacks a clear spatial problem-oriented approach and a transparent process for operationalizing variables [16].

Against this background, this study uses Shanghai as its empirical setting and addresses three research questions: 1) At the urban scale, which attributes of theme parks are significantly associated with positive emotions, and how are they ranked in terms of relative importance? 2) After controlling for multidimensional attributes, do these relationships exhibit differentiated patterns, including potential nonlinearity, threshold-based changes, or diminishing marginal returns? 3) At the urban spatial level, does the supply of theme parks exhibit significant clustering and hotspot patterns, and does emotional heterogeneity persist within these clustered areas? Accordingly, this study makes three main contributions. First, it establishes a comparable attribute–emotion measurement framework based on a multi-park urban sample. Second, it uses spatial analysis to explain why emotional outcomes differ across similar parks rather than treating spatial analysis as a supplementary visualization tool. Third, it combines interpretable machine learning with supplementary regression tests to distinguish robust nonlinear relationships from patterns that are merely visually suggestive.

## 2. Theoretical framework and literature review

Theme park experiences provide a classic example of a highly designed leisure-consumption context. Emotional responses not only reflect immediate experiences but may also translate into satisfaction and behavioral intentions; therefore, understanding the drivers of positive emotions holds clear theoretical and managerial significance [17]. Existing research in theme park settings has demonstrated that emotional dimensions such as pleasure and arousal can effectively characterize visitor experiences and are consistently correlated with outcome variables such as satisfaction [9]. At the same time, emotions are not triggered by a single attraction alone but are more likely shaped by multiple cues, such as thematic narratives, spatial environments, service interactions, and crowd dynamics. This implies that the sources of emotions must be discussed within a more comprehensive attribute framework [18]. However, in comparisons at the urban scale, merely highlighting the importance of emotions is insufficient; what is more critical is establishing a mechanistic framework capable of explaining the attribution of emotions, thereby providing interpretable pathways linking attribute differences to emotional differences.

At the theoretical level, tourism and leisure studies often conceptualize emotions as psychological states that can be characterized by specific dimensions, linking them to behavioral response mechanisms. Among these, the pleasure and arousal dimensions emphasized by the emotional circular model are most frequently used to explain emotional differences in high-intensity experiential settings [19]. Consequently, theme park research focuses not only on emotional levels but also on how emotions vary across spatial touchpoints and service processes, and attempts to attribute emotional differences to perceptible experiential cues [11]. However, when the scope of research expands from a single park to a multi-park sample at the urban scale, relying solely on dimensional descriptions is insufficient to explain “why systematic emotional differences exist between different parks”; a more explicit mechanistic framework is needed to link perceptual cues to emotional responses.

Attribution theory provides a direct pathway to understanding the aforementioned mechanisms. This theory posits that individuals’ explanations of event causes are typically characterized along three dimensions: locus of causation, stability, and controllability. Its core premise is that individuals provide causal explanations for event outcomes, and different attribution dimensions shape distinct emotional experiences and behavioral responses [20]. Among these, stability influences expectations regarding the repeatability of future outcomes, thereby affecting the persistence of emotional experiences. Furthermore, when individuals attribute positive outcomes to the intentional and controllable behaviors of others, they are more likely to evoke positive emotions such as gratitude, which in turn fosters social bonds and prosocial interactions [21]. In tourism contexts, tourists’ judgments of satisfaction with experiential outcomes and their emotional reactions are similarly influenced by attribution processes. In particular, when tourists interpret positive experiences as resulting from the controllable capabilities of the destination or service provider, they are more likely to form stable positive evaluations and subsequent behavioral tendencies [22]. Therefore, in this study, theme park attributes are regarded as external cues for tourists’ causal explanations. Based on the three-dimensional framework of attribution theory, this study classifies the key attributes according to the attributional characteristics they are most likely to reflect, as follows. First, IP strength and service quality align more closely with “external—relatively stable—controllable” cues; visitors are more likely to attribute positive experiences to the park’s design and operational capabilities, thereby generating stronger positive emotions such as immersion and satisfaction. Second, green-space leisure aligns more closely with “external—stable—low controllability” environmental cues, and is more likely to consistently enhance low-arousal positive emotions such as relaxation and pleasure. Third, structural conditions such as accessibility and crowding align more closely with “external—relatively stable—partially controllable” cues; their effects are more likely to alter visitors’ attribution judgments through time costs and waiting uncertainties, thereby generating nonlinear or marginal effect differences.

Existing research has identified experience cues that can be attributed at various levels. Servicescape theory suggests that physical environment and social interaction cues influence customers’ emotional responses and behavioral evaluations [14]. In theme park research, the association between servicescape elements and visitor experience quality and satisfaction has also been validated [15]. At the same time, thematic narratives and story worlds are considered key features that distinguish theme parks from general recreational spaces, capable of enhancing emotional engagement through immersion and identity formation [9]. Furthermore, both attention restoration theory and stress recovery research indicate that natural environmental characteristics can promote psychological recovery and positive emotions, demonstrating the independent value of natural and landscape elements in restorative experiences [23]. It is worth noting that external conditions such as transportation accessibility and congestion alter visitors’ time costs and the pace of their experiences, thereby influencing how they interpret emotional outcomes [11]. Such factors are particularly critical in comparative studies of theme parks at the urban scale, as they often reflect both urban spatial structures and travel chain constraints.

From a research paradigm perspective, studies on emotions in theme parks have long relied primarily on questionnaires and single-park case studies. This approach limits the generalizability of findings to the urban scale due to constraints related to sample scope and contextual differences [9]. In recent years, social media and online reviews have provided a new data foundation for capturing authentic experiences, and a research school centered on text mining and sentiment analysis has gradually emerged in the tourism field [24]. In theme park research, existing studies have begun to combine social media and geospatial analysis to visualize the distribution of emotions within parks, demonstrating the coupling relationship between emotions and spatial touchpoints [22]. However, overall, evidence demonstrating the simultaneous quantification of supply-side attributes, measurement of demand-side emotions, and spatial matching at the city scale—along with providing interpretable rankings of attribute contributions and evidence of nonlinear interaction patterns—remains relatively limited.

Based on the above review, this study adopts attribution theory as a mechanistic framework, treating various types of experience cues in theme parks as external cues that visitors can use for attribution. We examine the strength of their association with positive emotions and potential nonlinear relationships across a multi-park sample at the city scale [20]. Furthermore, considering the strengths of machine learning models in capturing complex relationships alongside their “black-box” risks, this study emphasizes maintaining a balance between prediction and interpretation. It employs interpretable methods to present the marginal contributions and threshold characteristics of key cues, thereby enhancing both theoretical testability and practical applicability [25].

## 3. Materials and methods

### 3.1. Study area and data sources

**Fig 1:**
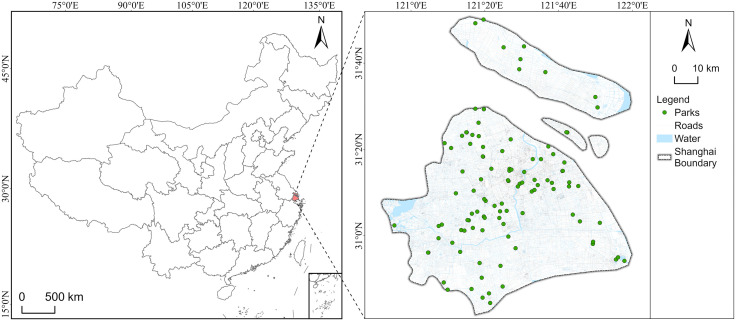
Map of Shanghai’s administrative districts and theme park distribution.

This study utilizes publicly available data and does not involve human subjects. All data collection complies with relevant laws, regulations, and platform terms of service. The study area is Shanghai, whose administrative boundaries and spatial distribution of theme parks are shown in Fig 1. There are a total of 106 theme parks in Shanghai, covering various types such as international brands and local parks. The primary data for this study include: ① Visitor review data: Ctrip (https://www.ctrip.com), China’s largest travel review platform, was selected as the primary data source [26]. Additionally, to enrich the dataset, this study concurrently collected popular posts and comments tagged with “Shanghai Theme Parks” from the Weibo (https://weibo.com) platform as supplementary data. Using the web crawling software Octopus Setup, visitor reviews for all theme parks in Shanghai on Ctrip and Weibo were collected, yielding approximately 15,0000 review entries. After data cleaning and preprocessing (removing HTML tags, links, and emojis, as well as deleting blank and duplicate reviews), a total of 144,360 valid reviews were retained for sentiment analysis. Each review includes the review text, posting time, and corresponding park ID. The data was scraped between December 15, 2025, and December 25, 2025, and the comment data covers the period from January 1, 2024, to November 15, 2025. ② Park Attribute Data: First, administrative boundaries and road network vector data for Shanghai were obtained from Natural Earth (http://www.naturalearthdata.com/). Then, Shanghai park POI data was retrieved via the Amap Open API (https://lbs.amap.com), from which 106 theme park POIs were filtered [27]. Using a Python script, we deduplicated and cleaned the POI names, coordinates, and other information, and extracted the geographic location and characteristic attributes of each park, including IP strength, stimulation level, green-space leisure, service quality, crowding level, ticket price, average daily visitor flow, and transportation accessibility indicators.

To enhance transparency and reproducibility, this study compiles all metrics used in subsequent models into a park-level analysis table. The overall positive emotion score (emotion_total) was calculated by aggregating and averaging the five positive emotions—joy, excitement, immersion, relaxation, and satisfaction—identified by BERTopic at the park level. Ticket price (ticket_price) directly records the park’s admission price. Facility count (facility_count) refers to the number of identifiable facilities or attractions within the park. Distance to the nearest subway station (distance_to_subway) was measured as the distance from the park centroid to the nearest subway station. The population within a 15-minute service area (service_population_15 min) was used to indicate the population size within a 15-minute service coverage area. IP strength (IP_strength), stimulation level (stimulation_level), green-space leisure (green_space_leisure), service quality (service_quality), crowding level (crowding_level), and accessibility score (accessibility_score) are park-level scoring metrics. These five metrics are derived from a comprehensive analysis of publicly available park descriptions, spatial environment data, and review texts regarding features related to thematic expression, stimulation, recreational environment, service quality, and perceived crowding. Initial values are then uniformly scaled linearly to a range of 0–10 to ensure comparability across different parks. The accessibility score combines accessibility information such as distance from subway stations and the population served, and is derived through directional adjustment and standardization to form a composite score; a higher value indicates better transportation accessibility and service coverage. The park size category (scale_category) classifies parks into small/medium, large, and extra-large categories based on national policies. The raw data, variable definitions, and park-level analysis tables used to reproduce the model results have been compiled into a minimal dataset for subsequent verification and replication. Table 1 summarizes the sample data overview. The number of theme parks in Shanghai is N = 106, with a total of N = 144,360 reviews. The mean number of reviews per park is 1,361.89 (SD = 1,603.26), and the median is 770. The distribution exhibits a long tail (with individual park reviews ranging from 200 to 11,790), indicating that a small number of popular parks attract a large volume of reviews.

**Table 1:** Sample overview.

Indicator	Value
Number of Parks (N)	106
Number of Reviews (N)	144,360
Average number of reviews per park	1,361.89
Reviews per park – SD	1,603.26
Reviews per park – median	770
Number of reviews per park - P25	440
Number of reviews per park - P75	1,785
Reviews per park – Minimum	200
Number of reviews per park – Maximum	11,790

### 3.2. Text sentiment quantification methods

A Transformer-based BERTopic model was employed to perform sentiment analysis on visitor review texts [28]. First, this study predefined five target positive emotions: joy, excitement, immersion, relaxation, and satisfaction, and prepared corresponding emotion keyword dictionaries based on these. The cleaned review corpus was input into the BERTopic model, which, supported by Chinese pre-trained BERT embeddings, automatically identified latent topics through UMAP dimensionality reduction and HDBSCAN clustering. Next, the cascaded TF-IDF algorithm was used to extract keywords for each topic, and these were combined with the emotion dictionary to determine the emotional category of each topic. The model identified a total of 25 topics, with each emotion category containing approximately 5 topics. Next, the probability distribution of each comment belonging to each emotional category was calculated, and these were aggregated at the park level to compute emotional scores. Specifically, for each park, the average probability of all comments across the five emotional categories was calculated to obtain scores for the five dimensions: pleasure, excitement, immersion, relaxation, and satisfaction. The average of these five scores was then taken as the park’s comprehensive positive emotion score (denoted as emotion_total, with a range of 0–1). This composite sentiment metric serves as the core dependent variable for subsequent analysis. Additionally, to verify the reliability of this metric, this study employs SnowNLP sentiment analysis to calculate sentiment polarity for the same set of comments. SnowNLP uses a Naive Bayes classification model to assign sentiment scores to Chinese text (0 indicates extremely negative, 1 indicates extremely positive) [29]. The SnowNLP sentiment score is calculated for each comment, and the park-level average is taken to obtain the park’s SnowNLP sentiment score (denoted as snownlp_pos_score). By comparing the BERTopic and SnowNLP sentiment scores, the consistency and validity of the text sentiment measurement can be verified.

### 3.3. Spatial analysis and park classification

Spatial statistical analysis was conducted using ArcGIS Pro software and Python libraries to reveal the spatial distribution patterns and emotional characteristics of theme parks. First, the kernel density estimation method was employed to identify spatial hotspots in park distribution. The bandwidth parameter was set to 1,500 meters (based on Silverman’s rule) [30], with a grid cell size of 50 × 50 meters. A citywide park density grid was calculated, and a kernel density heatmap was generated to visually illustrate the degree of clustering. Second, the Average Nearest Neighbor (ANN) index was employed to assess the spatial clustering of theme park locations [31]. The observed average proximity distance for all park locations was calculated and compared with the expected distance under a completely random distribution. An ANN ratio < 1 indicates a clustered distribution pattern, with significance tested using Z-scores and p-values. Third, the comprehensive positive sentiment scores of each theme park were mapped onto the spatial distribution map to examine whether theme parks exhibiting emotional heterogeneity still exist within the clustered areas. Finally, to facilitate a general description of the parks’ attribute configurations, this study performed K-means clustering (k = 3) based on five scoring criteria: IP strength, stimulation level, green-space leisure, service quality, and accessibility [32,33], and the cluster centers were provided for attribute profiling. As shown in Table 2, the three clusters identified are named: High Accessibility (highest score for transportation accessibility), Natural Recreation (higher scores for green-space leisure and IP attributes), and High-Quality Stimulation (outstanding scores for stimulation attractions and service quality). The average contour coefficient of the clustering is 0.183, indicating that the boundaries between groups are relatively blurred. Therefore, this study defines the results as attribute profiling groups for descriptive comparison and interpretive discussion, rather than as strong inferential evidence for typological classification. Subsequently, the average contour coefficient is used in the results section to evaluate clustering quality, ensuring a clear analytical distinction between the methodological process and the empirical findings.

**Table 2:** K-means (k = 3) clustering results and centroid scores (0–10).

Category ID	Category Name	n	Silhouette coefficient	IP strength	Stimulation Level	Green-space leisure	Service quality	Accessibility
0	High Accessibility	20	0.183	4.358	4.541	6.319	5.558	8.127
1	Nature and Leisure	39	0.183	6.699	4.123	7.065	5.815	3.801
2	High-Quality Stimulation	47	0.183	5.847	6.008	4.253	7.082	4.446

Note: Input features: IP strength, stimulation level, green-space leisure, service quality, accessibility. A higher silhouette coefficient indicates better separation between clusters.

### 3.4. Multivariate model

To evaluate the combined effects of theme park attributes on positive emotions, this study employs a random forest regression model to establish the relationship between emotion scores and various variables. Random forest is an ensemble learning method that constructs multiple decision trees through random sampling of samples and features, and improves predictive performance via voting or averaging. Compared to traditional linear regression models, random forests demonstrate significant advantages in both predictive accuracy and adaptability to complex data patterns [34]. This study uses the park’s comprehensive positive emotion score (emotion_total) as the dependent variable. The independent variables include 10 attributes: the park’s IP strength, stimulation level, green-space leisure score, service quality, and accessibility score, as well as ticket price, distance to the subway, population within a 15-minute service radius, number of facilities, and average crowding level (the top 10 variables ranked by feature importance). Model parameters were set to default values, and model performance was evaluated using 5-fold cross-validation. By calculating feature importance, this study measured the relative contribution of each variable to the model’s predictions. Two importance metrics were reported: Mean Decrease in Gini, based on the average reduction in the Gini index at decision tree nodes, and the more robust Permutation Importance (based on the impact of shuffling variable values on model error) [35]. The latter is considered to better reflect the true magnitude of feature effects because it eliminates the influence of units and multicollinearity. Both the training and validation of the random forest models were performed using Python’s scikit-learn library.

### 3.5. Formal statistical tests for nonlinear relationships

Given that PDP plots are primarily used to reveal the structural patterns within a model but are insufficient on their own to constitute statistical evidence of nonlinear relationships, this study conducted supplementary spline and segmented regressions at the park level. Using the comprehensive positive emotion score (emotion_total) as the dependent variable, this study included IP strength, service quality, and green-space leisure as core explanatory variables, while controlling for stimulation level, accessibility, admission price, distance from the subway, population within a 15-minute service radius, number of facilities, crowding level, and park size category. By comparing the fit of linear models, cubic spline models, and piecewise regression models, this study further examined whether significant nonlinear relationships exist among key variables, whether potential breakpoints exist, and how slopes change before and after these breakpoints. This analysis does not replace the random forest model but serves as supplementary validation of its interpretive results.

**Fig 2:**
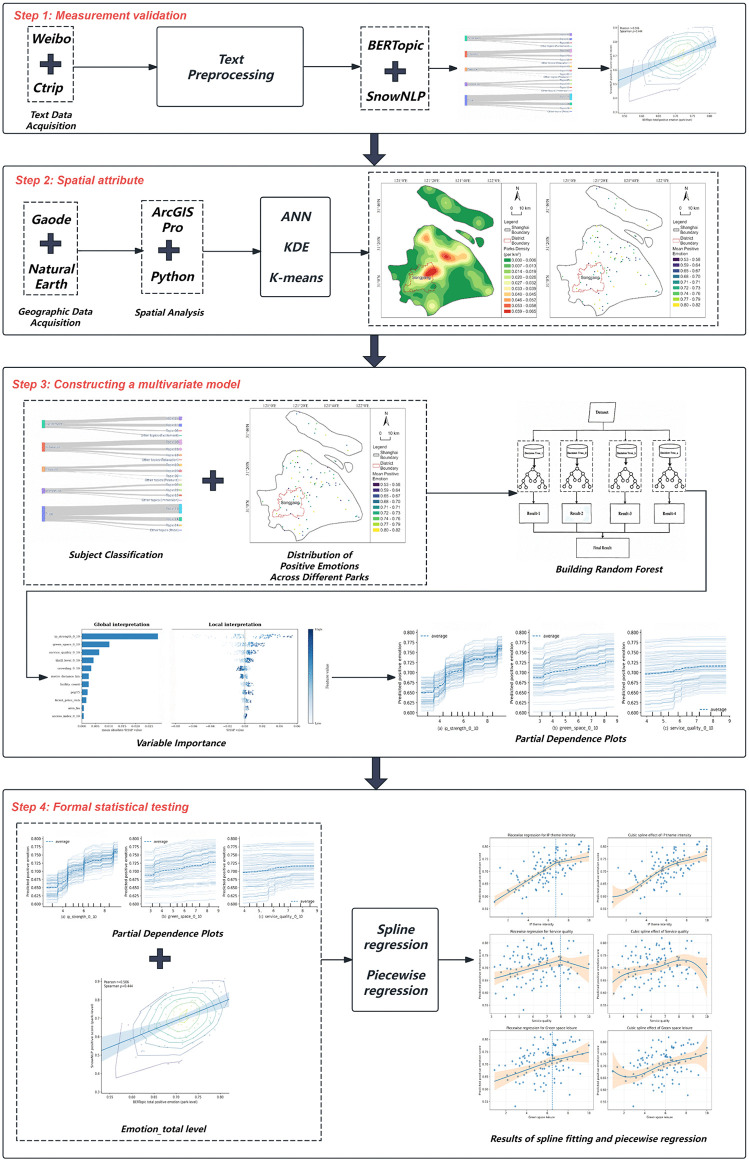
Research flowchart. Source: The map data in the figure is sourced from Natural Earth data.

Overall, as shown in Fig 2, the analytical workflow comprised four steps: 1) Quantification of positive emotions. 2) Analysis of theme park attributes and identification of spatial patterns. 3) Construction of a random forest model to identify the importance of key attributes and the nature of their relationships. 4) Formal statistical testing of nonlinear relationships among key variables using spline regression and piecewise regression.

## 4. Results

### 4.1. Descriptive statistics and park classification

The results of the sentiment analysis of visitor reviews for theme parks in Shanghai show that the comprehensive positive sentiment scores for all parks range from 0.531 to 0.820, with a mean of approximately 0.709 (SD = 0.058). The percentile distribution of sentiment was P25 = 0.675, the median was 0.712, and P75 = 0.747, indicating that most parks exhibited high levels of positive sentiment with moderate dispersion [36]. A summary of descriptive statistics for the five positive sentiment dimensions and SnowNLP sentiment scores is presented in Table 3. Significant differences in scores across different emotional dimensions were observed at the park level: the pleasure dimension had the highest average (0.803), followed by excitement (0.763), with satisfaction and immersion in the middle (0.742 and 0.667, respectively), and relaxation having the lowest average (0.571). The pleasure and satisfaction dimensions both had high means and relatively large standard deviations, indicating more pronounced differences among parks in these two dimensions. The relaxation dimension exhibited the highest dispersion (SD = 0.174), indicating greater variation among parks in the expression of positive emotions related to restful environments. The park-level mean for SnowNLP sentiment comparison scores was 0.696, slightly lower than the overall positive sentiment mean of 0.709, but the distribution patterns of the two were generally similar.

**Table 3:** Descriptive statistics of sentiment scores (0–1).

Variable	Mean	Standard Deviation	Minimum	P25	Median	P75	Maximum
Overall positive emotions	0.709	0.058	0.531	0.675	0.712	0.747	0.82
Pleasure	0.803	0.104	0.33	0.747	0.822	0.879	0.955
Excitement	0.763	0.115	0.448	0.711	0.787	0.829	0.948
Immersion	0.667	0.135	0.338	0.592	0.666	0.765	0.939
Relaxation	0.571	0.174	0.108	0.448	0.584	0.698	0.884
Satisfaction	0.742	0.093	0.492	0.681	0.75	0.821	0.906
SnowNLP benchmark score	0.696	0.11	0.293	0.629	0.694	0.774	0.917

Note: P25/P75 refers to the quantiles.

To compare emotional differences across different theme parks, this study grouped the park samples based on the clustering results obtained in Section 3.3 (Table 2) and conducted a one-way ANOVA. The mean and standard deviation of the overall positive emotion scores for each park category are shown in Table 4. Descriptive comparisons based on attribute profiles indicate that there are differences in the mean overall positive emotion scores across different groups. The mean for the Nature and Leisure group was 0.726 (SD = 0.055), 0.686 (SD = 0.062) for the High Accessibility group, and 0.705 (SD = 0.056) for the High-Quality Stimulation group. A one-way ANOVA revealed statistically significant differences between groups (F(2,103) = 3.485, p = 0.034, η² ≈ 0.063). Given that the clustering coefficient was 0.183 and the group boundaries were weak, the above test results are primarily presented as exploratory descriptions to indicate potential differences in attribute configurations, rather than as validation of a stable typological structure [35].

**Table 4:** Comprehensive positive emotion scores and comparative analysis of differences across different types of theme parks.

Theme Park Type	n	Mean	Standard Deviation	df (between groups)	df (within-group)	F	p	η²
Nature and Leisure	39	0.726	0.055	–	–	–	–	–
High Accessibility	20	0.686	0.062	–	–	–	–	–
High-Quality Stimulation	47	0.705	0.056	–	–	–	–	–
Clustering Types (3 types)	–	–	–	2	103	3.485	0.034	0.063

In addition to thematic positioning, the size of a park may also influence visitor emotions. Based on the “Guiding Opinions on Regulating the Construction and Development of Theme Parks (Development and Reform Commission Social Regulation [2018] No. 400)” [37], this study classifies Shanghai’s theme parks into three categories: extra-large, large, and small-to-medium. A comparison of emotional differences is presented in Table 5. The results indicate that extra-large parks had the highest average positive emotion (0.760, SD = 0.064), followed by large parks (0.727, SD = 0.050), with small and medium-sized parks having the lowest (0.702, SD = 0.057). The results of the one-way ANOVA indicate a significant main effect of park size (F(2,103) = 3.933, p = 0.023, η² = 0.071), with emotional scores increasing as park size increases. The effect size for park size is slightly higher than that for park type, suggesting that larger theme parks generally provide visitors with a more intense positive emotional experience [38]. The reasons for this include the fact that large parks typically offer a richer array of facilities, more expansive spaces, and more abundant service resources, making it easier for visitors to experience surprise and satisfaction; simultaneously, large scale may also imply greater brand appeal and quality assurance, thereby enhancing visitors’ psychological expectations. In subsequent analyses, this study will examine the independence of the scale effect after controlling for confounding factors such as location and IP influence.

**Table 5:** Comprehensive positive emotion scores and comparative analysis of differences across park size categories.

Theme Type	n	Mean	Standard Deviation	df (between groups)	df (within-group)	F	p	η²
Small and medium-sized	84	0.702	0.057	–	–	–	–	–
Large	16	0.727	0.050	–	–	–	–	–
Extra-large	6	0.760	0.064	–	–	–	–	–
Scale Level (Level 3)	–	–	–	2	103	3.933	0.023	0.071

In contrast, differences in scale classifications based on policy standards exhibited higher reproducibility. The mean sentiment scores for extra-large, large, and small-to-medium-sized parks all showed an upward trend, and analysis of variance (ANOVA) revealed a significant main effect (F(2,103) = 3.933, p = 0.023, η² = 0.071). Therefore, this study adopts size grouping as a more reproducible external comparison, while K-means clustering, due to its weaker clustering quality, should only be regarded as a descriptive sensitivity test.

### 4.2. Measurement validity and methodological consistency

After quantifying emotions using the BERTopic model, this study examined the validity and stability of this metric. First, Table 6 presents the results of grouping the 25 topics identified by BERTopic into emotional categories. Among the five positive emotion categories, satisfaction had the highest overall prevalence (∑ prevalence = 0.356), far exceeding the other categories; relaxation ranked second (0.215), excitement ranked in the middle (0.187), while joy and immersion had the lowest prevalence (0.121 each). This indicates that among online reviews of Shanghai theme parks, content expressing satisfaction has the highest proportion, followed by relaxation and excitement, while direct expressions of joy, pleasure, or immersion are relatively scarce. This phenomenon may reflect that visitors tend to emphasize experiences that highlight identity and quality recognition in their reviews, while mentioning general feelings of happiness, satisfaction, or immersion in less detail. Table 6 also lists the top three representative themes and their keywords for each emotion category. For example, in the “Excitement” category, Theme #16—which accounted for the highest proportion (7.8% of all reviews)—included keywords such as “speed, thrill, night rides, challenge, roller coaster, heart-pounding,” indicating that many visitors experienced excitement and thrills through nighttime roller coaster rides and similar attractions. In the “Relaxation” category, the largest theme, #08 (accounting for 11.3%), includes terms such as “slow pace, greenery, scenery, strolling, comfort, and lake,” reflecting the relaxing experience of leisurely walks and connecting with nature in urban parks. These thematic keywords provide clues for emotional attribution: excitement stems primarily from thrilling attractions and challenges, satisfaction is linked to the scale and quality of the park, and relaxation is attributed to the environment and pace. The keywords in these categories generally align with the definitions established in this study, supporting the content validity of the BERTopic model results.

**Table 6:** Summary of theme-emotion category mapping.

Emotional Category	Category Prevalence (Σprevalence)	Representative Themes (Top 3)
Excitement	0.187	16-Theme 16-Excitement (0.078): Speed, Thrill, Nightlife, Challenge, Attractions, Roller Coaster, Heart-pounding 11-Theme 11-Excitement (0.064): Screams, Speed, Nighttime, Thrill, Excitement, Roller Coaster, Waiting in Line 06-Theme 06-Excitement (0.025): Nightlife, Thrills, Challenges, Queues, Screams, Rides, Heart-pounding
Pleasure	0.121	10-Theme 10-Pleasure (0.053): Experience, Happy, Satisfied, Atmosphere, Family-friendly, Friendly, Service 20-Theme 20-Pleasure (0.051): Friendly, Satisfied, Clean, Recommended, Happy, Service, Experience 00-Theme 00-Pleasure (0.011): Family, Service, Happy, Fun, Recommend, Experience, Satisfaction
Relaxation	0.215	08-Theme 08-Relaxation (0.113): Slow pace, greenery, scenery, walking, shade, comfort, lake 23-Theme 23-Relaxation (0.055): Air, Lake, Greenery, Slow Pace, Shade, Strolling, Comfort 18-Theme 18-Relaxation (0.035): Slow pace, Greenery, Scenery, Air, Lawn, Strolling, Lake
Immersion	0.121	07-Theme 07-Immersion (0.040): IP, Parade, Immersion, Set Design, Characters, Photo Opportunities, Storyline 22-Theme 22-Immersion (0.037): Storyline, Photo-taking, Performance, Interaction, Theme, Set Design, IP 12-Theme 12-Immersion (0.031): Set design, storyline, characters, performances, interaction, immersion, theme
Satisfaction	0.356	19-Theme 19-Satisfaction (0.218): Top-tier, Scale, Quality, City’s Calling Card, Culture, Impressive, Safety 14-Theme 14-Satisfaction (0.088): Scale, Impact, City’s Calling Card, Quality, Culture, Satisfaction, Domestic 24-Topic 24-Satisfaction (0.033): Management, Satisfaction, Quality, Impact, Culture, Scale

**Fig 3:**
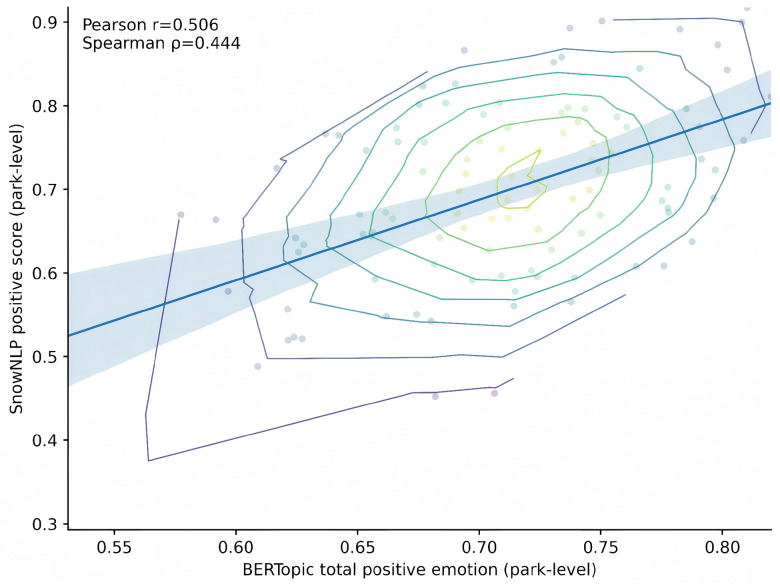
Consistency between BERTopic and SnowNLP.

Subsequently, this study examined the consistency between BERTopic sentiment scores and SnowNLP scores. Calculated at the park level, the Pearson correlation coefficient between the sentiment scores obtained by the two methods was 0.506, and the Spearman correlation was 0.444, both reaching a moderate level [39,40]. See Fig 3 for details. This indicates that the sentiment indices extracted by BERTopic are generally consistent with those of traditional sentiment analysis methods in terms of overall trends, supporting the use of “park-level positive sentiment scores” as a reliable analytical indicator in this study. In contrast, at the comment level, the correlation coefficients between the two methods were only approximately 0.17 (Pearson) to 0.16 (Spearman), indicating weaker consistency. This implies that for individual short comments, discrepancies often arise between BERTopic topic probabilities and SnowNLP sentiment scores, likely due to differences in judgment caused by sarcasm, humor, or missing context. However, when comments are aggregated to the park-level average, these random errors partially offset each other, resulting in a more robust metric. Therefore, this study adopted a “BERTopic-primary, SnowNLP-secondary” strategy: using BERTopic sentiment scores as the primary analysis metric, while employing SnowNLP results to verify convergence and identify outliers.

Furthermore, this study compared the differences between the two sentiment calculation methods across the results of each park to identify potential data anomalies. A threshold was set such that the absolute difference between a park’s BERTopic sentiment score and its SnowNLP sentiment score must be > 0.20. The results revealed that three park samples exceeded this threshold, as listed in Table 7. For example, the emotion total for Park P106 was 0.706, while the SnowNLP score was only 0.456, a difference of 0.25. Similarly, the difference between the two sentiment scores for P002 and P018 also exceeded 0.23. These outliers suggest that reviews for parks such as P106 may exhibit unique emotional expression patterns, such as extensive use of sarcasm or statements that separate positive and negative sentiments, leading to significant discrepancies in emotional scores between the two algorithms. The authors conducted a case-by-case review of the anomalous parks and found that P106 involved a newly opened park where positive praise and negative complaints coexisted in the reviews, resulting in a marked difference in emotional scores between the two algorithms. For such samples, caution should be exercised in subsequent analyses, and attention should be focused on the areas for improvement in these parks. It should be noted that this study did not exclude any parks due to differences in sentiment metrics; the outlier analysis was primarily used to verify the traceability and robustness of the results.

**Table 7:** List of outliers (threshold > 0.20).

park_id	park_name	cluster_k3	scale_ category	Emotion_total	snownlp_ pos_score	Absolute difference	n_ comments
P106	Shanghai Theme Park 106	High-Quality Stimulation	Small and medium-sized	0.706	0.456	0.25	37
P018	Shanghai Theme Park 18	High-Quality Stimulation	Small and medium-sized	0.531	0.293	0.238	61
P002	Shanghai Theme Park 2	Nature and Leisure	Large	0.682	0.453	0.23	33

Note: Anomaly threshold: |emotion_total - snownlp_pos_score| > 0.20.

### 4.3. Spatial pattern analysis

**Fig 4:**
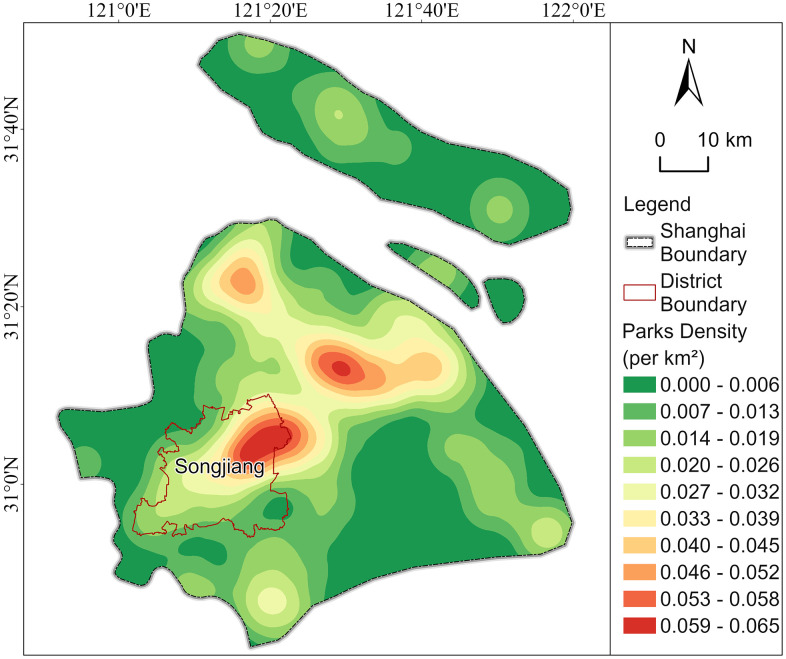
Kernel density heatmap of the spatial distribution of themed parks in Shanghai. Note: Redder colors indicate higher park density; the figure highlights the primary clustering area in Songjiang District.

**Fig 5:**
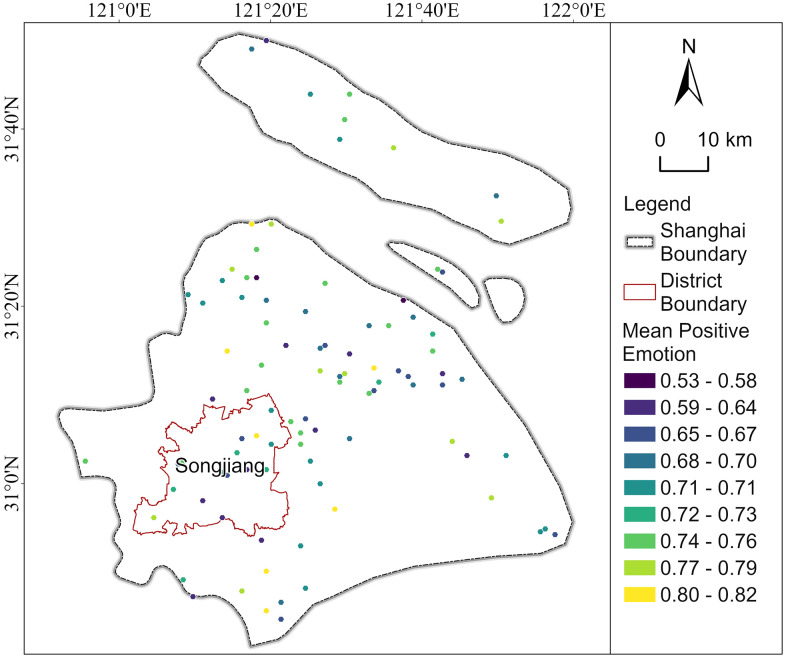
Distribution map of theme park clusters and positive visitor sentiments in Shanghai.

The geographic distribution of Shanghai’s theme parks was analyzed using the average nearest neighbor test, yielding an ANN of 0.443, significantly lower than 1 (z = −10.964, p < 0.001). This indicates a significant deviation from a completely random distribution and reveals a clustered spatial pattern. The observed average nearest neighbor distance was approximately 1.56 kilometers, significantly shorter than the random expected distance of 3.518 kilometers. The kernel density heatmap (Fig 4) identifies Songjiang District as the primary hotspot area, addressing the question of where parks are primarily concentrated. However, when the overall positive emotion scores for each park are overlaid on the spatial distribution map (Fig 5), distinct variations in emotional levels can still be observed even within the clustered areas. This further addresses the question of whether emotions are consistent within clustered areas. This heterogeneity within clusters indicates that while spatial proximity reflects supply concentration and urban functional zoning, it does not necessarily imply similar emotional outcomes. In summary, ANN is used to identify the presence of clusters, KDE is used to locate hotspot structures, and the park-level emotion overlay map is used to examine whether emotional heterogeneity exists within hotspots.

### 4.4. Analysis of random forest model results

**Fig 6:**
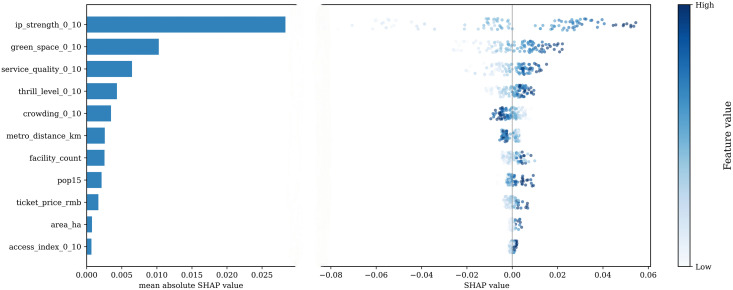
Permutation importance of park attributes.

**Fig 7:**
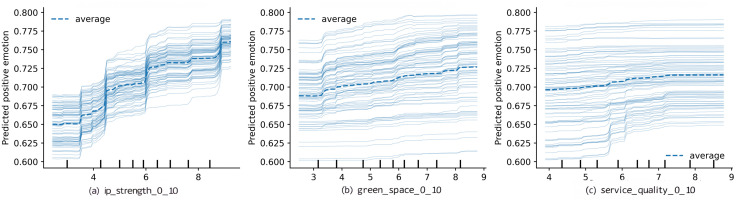
Partial dependence plots for key park attributes and predicted positive emotion.

Within the framework of multivariate analysis, the explanatory power of park attributes on positive emotions is moderate. The random forest model, validated using 5-fold cross-validation, yielded an average coefficient of determination R² = 0.433 (standard deviation SD = 0.128), root mean square error RMSE = 0.042, and mean absolute error MAE = 0. 034. This indicates that the model explains approximately 43.3% of the variance in positive emotions at the park level; however, a significant proportion of residual variance remains, which may reflect unaccounted-for factors such as time, management processes, visitor composition, and situational factors. Fig 6 displays the feature contributions based on permutation importance, providing a visual comparison of the relative importance of each variable. Among all candidate variables, the importance of IP strength was significantly higher than that of other variables, reaffirming its dominant role. In contrast, variables related to park size and accessibility did not rank among the top in terms of importance. This does not imply that they are entirely irrelevant; rather, it likely indicates that their effects primarily manifest in altering visitation conditions, crowding pressure, and experiential context, rather than directly constituting the dominant source of emotional differences between parks, as IP strength does. Fig 7 presents partial dependence plots illustrating the relationships between (a) IP strength, (b) green-space leisure, and (c) service quality and predicted positive emotion scores. These PDP plots show that IP strength exhibits a distinct nonlinear pattern, green-space leisure displays an overall monotonically increasing positive correlation, while service quality may show a potential change in slope across higher values.

### 4.5. Regression analysis

However, visual inspection alone is insufficient to confirm nonlinear structures; therefore, this study conducted supplementary spline regression and piecewise regression estimates, with the results summarized in Table 8. As shown in the table, formal tests of the spline and piecewise regression results indicate that, among the three core variables, only the nonlinear relationship of IP strength received robust statistical support. Specifically, the spline test results for IP strength were significant (F = 3.217, p = 0.027). Segmented regression further identified a potential inflection point at approximately 6.730, and the segmented model showed significant improvement over the linear model (F = 10.975, p = 0.001), indicating a relatively clear nonlinear relationship between IP strength and positive emotion. Combining the changes in slope before and after the inflection point, it can be further inferred that the positive association between IP strength and positive emotions is stronger at low to moderate levels, while its positive marginal effect weakens once this level is exceeded. In contrast, although service quality showed a possible inflection trend around 7.963, neither the spline test (F = 1.569, p = 0.202) nor the segmented test (F = 3.098, p = 0.082) reached statistical significance. Therefore, it is more appropriate to regard this as an exploratory finding rather than drawing firm conclusions regarding a stable threshold or saturation effect. Similarly, the spline test (F = 0.670, p = 0.572) and the segmented test (F = 0.260, p = 0.611) for green-space leisure were both non-significant, indicating that the relationship between green-space leisure and positive emotions is generally closer to a stable positive association rather than a nonlinear relationship with a clear inflection point.

**Table 8:** Tests for nonlinear patterns.

Predictor Variables	Spline F-value	Spline p-value	Segment Point	Segment F	Segment p	Inference
IP strength	3.217	0.027	6.730	10.975	0.001	Supports nonlinearity; the positive slope after the inflection point is weak.
Service Quality	1.569	0.202	7.963	3.098	0.082	Possible turning point, but not statistically significant.
Green-Space Leisure	0.670	0.572	6.475	0.260	0.611	Overall positive correlation; no stable breakpoints.

Note: The spline model tests whether a nonlinear specification fits the data better than a linear specification. The segmented model estimates candidate breakpoints and the slopes before and after each breakpoint.

**Fig 8:**
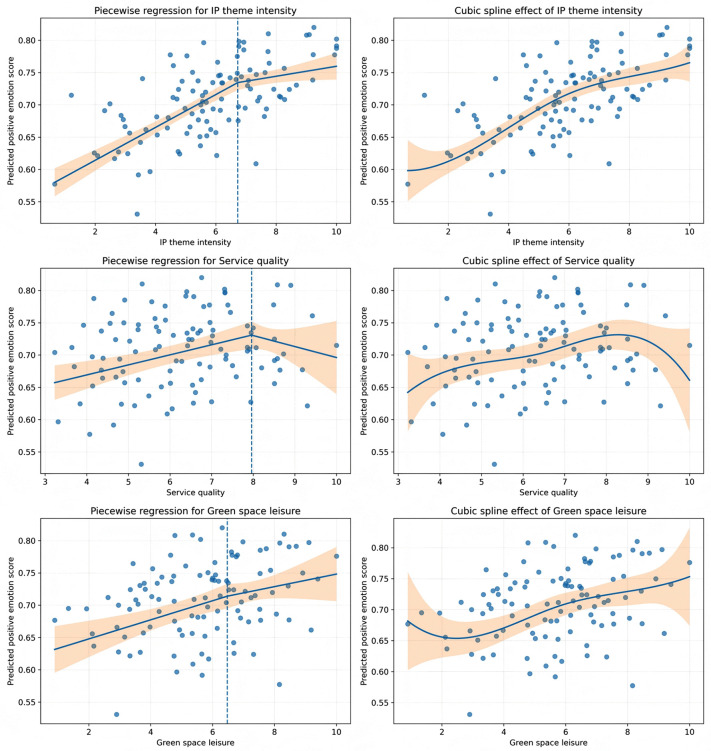
Results of spline fitting and piecewise regression.

Fig 8 further illustrates the relationship patterns among the three types of variables within the spline fitting and piecewise regression frameworks. Judging from the graphical trends, the fitting curve corresponding to IP strength rises rapidly before reaching the intermediate level and gradually flattens out as it approaches the breakpoint, consistent with the statistical conclusion shown in Table 8: “Supports nonlinearity; the positive slope after the inflection point is weak.” Although the fitting curve for service quality exhibits some changes in the high-value range, the overall confidence interval is relatively wide, and formal statistical tests did not reach the significance level, indicating that its nonlinear pattern lacks robust support. The fitting curve for green-space leisure, on the other hand, exhibits a generally stable positive trend without showing any clear or stable structural inflection points. Therefore, based on both the visual inspection and the results of formal statistical tests, this study cautiously concludes that the nonlinear characteristics of IP strength are strongly supported, while the related nonlinear patterns for service quality and green-space leisure should primarily be regarded as exploratory trends.

## 5. Discussion and conclusions

### 5.1. Discussion

Previous studies have shown that emotional experiences in theme parks can be characterized by dimensions such as pleasure and arousal, which further influence satisfaction and behavioral intentions [3,9]. However, emotions do not stem solely from the attractions themselves, but rather from a comprehensive experience triggered by the overall environment and brand perception [41]. Overall, the results indicate that in Shanghai’s theme parks, positive emotions are more strongly associated with perceptible experiential cues than with spatial distance alone. Random forest and permutation importance analysis consistently identified IP strength, service quality, and green-space leisure as the most informative predictors, while supplementary spline regression and segmented regression revealed that only IP strength exhibited a statistically significant nonlinear relationship. Specifically, the nonlinearity test for IP strength was significant (F = 3.217, p = 0.027), with an estimated inflection point of approximately 6.73. In contrast, the corresponding nonlinear patterns for service quality and green-space leisure did not receive statistical support. These results refine the interpretation of the PDP plots. Threshold-like nonlinear patterns appear robust only for IP strength, whereas patterns for service quality and green-space leisure should be considered exploratory rather than definitive. Thus, positive emotions in theme parks appear to be shaped by strong thematic signals, service process quality, and restorative environmental cues, though not all of these cues operate through the same functional mechanisms.

IP strength ranked first in predicting differences in visitors’ positive emotions. This result suggests that in highly designed leisure environments such as theme parks, thematic narratives and symbolic cues are more closely associated with emotional expression than many other supply-side attributes. This aligns with the earlier characterization of IP themes as “external, controllable, and relatively stable” cues. Unified thematic narratives, scene consistency, and character symbols make it easier for visitors to attribute positive feelings to the park’s own design and operational capabilities, thereby fostering more enduring emotional memories and relational bonds. This process resembles the “sensory–emotional–cognitive–behavioral” response chain emphasized in brand experience theory [42] and aligns with the mechanisms of identity connection and satisfaction generation identified in consumer–organizational identity research [43]. Furthermore, supplementary spline regression and piecewise regression provide clearer statistical support for the nonlinear nature of this relationship. The breakpoint estimate is approximately 6.73, and the positive slope after the inflection point is significantly weaker than before it. Therefore, a more reasonable interpretation is not that IP strength continues to accelerate after the inflection point to drive emotional elevation, but rather that thematic coherence is most strongly associated with positive emotional elevation at low to moderate levels. Once thematic expression reaches a high level, its continued enhancement remains positively correlated with positive emotions, but the marginal returns tend to diminish. In other words, IP strength does not exhibit a continuous acceleration where higher levels lead to faster growth, but rather a structural pattern in which positive gains slow down after exceeding a certain level. It is important to emphasize that this study reveals statistical associations and nonlinear patterns; mechanistic explanations should still be regarded as reasonable inferences based on attribution logic and narrative research, rather than direct proof of causality.

In contrast, the revised evidence regarding service quality is more cautious. Although the PDP suggests a possible inflection point at higher service levels, neither the cubic spline comparison nor the piecewise regression provided strong statistical support for a stable threshold or saturation effect. Therefore, service quality should primarily be interpreted as an overall positive process indicator rather than a variable with an established precise threshold. However, this does not diminish its managerial significance; queue transparency, orderly guidance, and incident response continue to influence visitors’ evaluations of the process, particularly in crowded and time-constrained environments. Recent research suggests that real-time crowding information can influence tourists’ schedule adjustment and destination choices, especially under tighter time constraints, while improved crowding communication may also help visitors adjust expectations and mitigate the negative experiential consequences of congestion [44].

The role of green-space leisure is better understood as a broad positive association rather than a sharp nonlinear relationship. Formal tests did not reveal statistically significant nonlinear improvements for green-space leisure, nor did segmented regression provide evidence of stable inflection points. Therefore, it is more reasonable to understand green and restorative spaces as stable emotional recovery resources that promote relaxation and pleasure across various park conditions, rather than as attributes that trigger discrete threshold effects. This interpretation is consistent with attention recovery theory and aligns statistical conclusions with the evidence [23]. Unlike the high arousal induced by thrill rides, green-space leisure is more likely to enhance low-arousal positive emotions such as relaxation and pleasure, amplifying their marginal value in the context of high-density urban living; thus, the role of natural elements in theme parks should not be reduced to mere landscape embellishment. The findings of this study further support a rhythmic experience explanation: high-arousal rides, waiting in lines, and social interactions accumulate psychological load, while accessible green-space leisure segments provide visitors with windows for emotional reset, thereby enhancing overall positive emotion levels. This explanation also offers a pathway for understanding emotional differences across park types: nature-oriented parks are more likely to sustain positive emotions through restorative experiences rather than relying on short-term high stimulation.

Furthermore, in this study, parks with high accessibility did not exhibit the highest overall positive emotions, and Fig 6 also indicates that variables related to park size and accessibility are not particularly significant. This result suggests that, in comparisons at the park level, location and scale are more likely to function as contextual conditions or structural constraints rather than primary factors directly determining emotional differences. Their effects are often indirectly manifested through crowding pressure, visit convenience, time costs, and visitor expectations. Therefore, from a management perspective, the key lies not in simply increasing accessibility or expanding scale, but in mitigating the crowding externalities caused by convenience and agglomeration through clearer information provision, spatiotemporal flow management, and more predictable process management.

From a methodological perspective, the combination of average nearest-neighbor analysis, kernel density estimation, and sentiment overlay maps offers complementary value in this study. Average nearest-neighbor analysis is used to determine whether agglomeration exists at the urban scale, kernel density estimation is used to identify hotspot locations, and sentiment overlay maps are used to examine whether sentiment consistency exists within these hotspots. This combination allows the study to advance from the question of “where agglomeration occurs” to “whether similar sentiment patterns emerge within agglomeration zones,” thereby helping to truly integrate spatial analysis into the explanatory framework. Related studies provide methodological support for the relationship between crowding, sentiment, and satisfaction, and also emphasize the situational dependence of crowding experiences [45,46]. This interpretation aligns with a recent review study, which indicates that although such effects remain context-dependent, perceived crowding is generally associated with weaker emotional and evaluative outcomes in tourism [47]. However, this set of methods is still primarily used to identify spatial patterns and heterogeneity cues and cannot, on its own, prove spatial spillover or causal relationships. At the same time, current research has not yet incorporated visitor trajectories, actual visit durations, and changes across multiple time periods into the analysis; therefore, the explanatory power of spatial methods remains limited by the constraints of cross-sectional data.

### 5.2. Recommendations

These findings yield practical implications in two areas. First, at the park level, managers should prioritize interventions that are directly perceptible to visitors across multiple touchpoints. For parks with weak thematic coherence, investments that strengthen narrative cohesion among attractions, performances, signage, staff appearance, and commercial spaces are often more effective than simply adding isolated thematic elements. Regarding services, the focus should not be on indiscriminately improving all metrics, but rather on enhancing reliability in areas of high uncertainty—particularly queue communication, real-time information, crowd guidance, and incident management. For green-space leisure, the implication is not the need for a single dramatic environmental upgrade, but rather the incorporation of dispersed rest nodes throughout the entire visitor journey.

Second, at the urban management level, spatial analysis results indicate that planning should transcend simple hotspot logic. Since parks within the same cluster may exhibit significant differences in emotional experience, investment and governance should not be based solely on geographic location. In densely clustered areas such as Songjiang District, traffic coordination, visitor flow monitoring, and staggered event scheduling may help mitigate the externalities caused by congestion. Outside core hotspot areas, providing more small-scale, mood-restorative recreational facilities helps broaden the geographic coverage of urban recreational needs. Furthermore, given the weak separation revealed by the K-means clustering analysis, the park types identified in this study are best regarded as descriptive features rather than a strict classification system. Therefore, the above recommendations are positioned as cross-sectoral priorities rather than mandatory regulations for rigid park categories.

### 5.3. Limitations and future directions

The following limitations should be noted. First, the random forest model explained 43.3% of the variance in positive emotions, indicating that while its explanatory power is meaningful, it is not exhaustive. While this result is reasonable for park-level emotional studies, it also suggests that some important sources of variation have not been accounted for, including weather, seasonality, special events, wait times, price promotions, companion composition, and sociodemographic differences among visitors. Future research could enhance explanatory power by integrating variables related to time, behavior, and management processes. Second, although the park-level attribute framework improves comparability, variables related to experiential evaluation—such as immersion intensity, service quality, and green-space leisure—remain constrained by operational definitions and aggregation choices. More transparent coding guidelines, inter-rater reliability checks, and external validation will enhance the study’s reproducibility. Third, the K-means clustering method has a low silhouette coefficient, so the clustering results should be viewed as heuristic groupings of characteristics rather than robust inferential categories; this is also why this study avoided drawing theoretical conclusions based solely on ANOVA. Fourth, the supplementary nonlinear tests were conducted only on 106 parks; while this aligns with the current study design, it limits the statistical power to detect more subtle functional forms. Finally, although spatial analysis effectively identifies clustering and hotspot heterogeneity, it cannot establish causal spatial spillover effects. Multi-city panel data, longitudinal retrospective studies, and visitor trajectory data would help test whether the observed patterns are stable across different times and contexts.

### 5.4. Conclusion

In summary, this study demonstrates that positive emotions in Shanghai’s theme parks are jointly shaped by experiential attributes and the urban spatial context, though not all of these relationships are linear. IP strength is the most influential attribute and the key predictor for which statistical support for nonlinearity was obtained through supplementary tests; its marginal association exhibits a decreasing trend after reaching moderate to high levels. Service quality and green-space leisure remain important positive correlates, but their nonlinear forms have not yet been confirmed and should therefore be interpreted with caution. At the spatial level, theme parks exhibit a significant clustered distribution, yet positive emotions vary within the same hotspot area, indicating that clustering does not necessarily lead to emotional similarity. Essentially, this study provides a more cautious and evidence-based interpretation of the relationship between theme park attributes and positive emotions at the urban scale. Methodologically, this study demonstrates the value of combining text-derived sentiment indicators, spatial statistics, interpretable machine learning, and supplementary tests of functional form, thereby enabling the distinction between robust nonlinear relationships and patterns that are merely visually suggestive.

## Supporting information

S1 File Minimal dataset used for analysis. (XLSX)

S2 File Includes Jupyter Notebook code containing reproducible analysis results. (IPYNB)

S3 File Excel file containing data values used to generate figures. (XLSX)
