# Supplementary material for: How do theme park attributes influence visitors’ positive emotions? Nonlinear effects and spatial heterogeneity in Shanghai’s theme parks
Source: PLoS One. 2026 Jun 18;21(6):e0344051. doi: 10.1371/journal.pone.0344051 (PMC13278401; doi:10.1371/journal.pone.0344051)
Supplement: Attachment — Submitted filename: REVIEWER PONE-D-26-07392.pdf [file pone.0344051.s004.pdf]

REVIEWER - Article PONE-D-26-07392

How Do Theme Park Attributes Influence Visitors' Positive Emotions? Nonlinear Effects and Spatial Heterogeneity in Shanghai's Theme Parks

The study analyzes theme parks in Shanghai, aiming to quantify visitors' positive emotions and identify associated attributes. To achieve this, it integrates online analytics, POI data and geospatial information, conduct spatial pattern analysis and develop interpretable predictive models. But perhaps these spatial analyzes have been little explored in the thematic figures presented and in the discussion of their efficiency.

Therefore, we suggest some revisions to the text and figures presented.

Comments

- Hi topics 1 (Introduction) and 2 (Theoretical Foundations and Literature Review) do not reflect the spatial methodological approach described in the Methodology and presented in the Results. The emphasis is on "positive emotions" related to tourism and nothing on the spatial approach. It is practically by summarizing the ideas presented that, without references, addresses that: "*In summary, this study employs Shanghai as its empirical setting, integrating text sentiment quantification, spatial pattern analysis, and multivariate modelling to deeply analyse the influence mechanisms and differential effects of various theme park attributes on visitors' positive emotions.*";
- How do theme park attributes influence visitors' positive emotions? or What is the influence of theme park attributes on visitors' positive emotions?
- review the structure of the text, the paragraphs are very long and contain some repeated questions. For example, there are paragraphs in the Results that practically take up an entire page;;
- in Table 2 in item 3.3, I suggest standardizing GS or GSR in relation to green spaces;
- I suggest that the text in item 3.3 "*The average contour coefficient for clustering was 0.183, indicating relatively blurred group boundaries.*" be presented in results and not in methodology;
- In general, references are not updated. Only 10 of the 48 references are from after 2020;
- I suggest increasing the text font in Figure 2 (Step 3 multivariate model);
- Figures 1, 4 and 5 make no mention of the location of the Pudong New Area and Songjiang District. I suggest including these locations on the map, as they are important areas of spatial concentration of tourism activities and will allow you to follow the authors' analyses;
- I have the impression that the analysis of Figures 4 and 5 is not compatible with the text presented and that the order of these figures is wrong;

- It seems that Figure 4 does not allow the map to read, as described in item 4.3, the spatial relationships between park sizes and the average of positive emotions;
- Apparently Figure 6 shows how little influence the "area" has on "Importance contributions";
- Figure 1 shows, in Shanghai, the parks and the road network (perhaps in terms of accessibility, but a topic little covered in the analysis). However, no map presents green spaces, a relevant topic in the analysis of topic 4.4 of the Results and at the beginning of the Discussion and Conclusions;
- I highlight that nothing in the Discussions, Recommendations and Conclusions was addressed regarding the approach and efficiency of the methodology used in spatial analysis.
