# Supplementary material for: How do theme park attributes influence visitors’ positive emotions? Nonlinear effects and spatial heterogeneity in Shanghai’s theme parks
Source: PLoS One. 2026 Jun 18;21(6):e0344051. doi: 10.1371/journal.pone.0344051 (PMC13278401; doi:10.1371/journal.pone.0344051)
Supplement: Attachment — Submitted filename: REVIEWER - R1 - PONE-D-26-07392.pdf [file pone.0344051.s006.pdf]

REVIEWER - Article PONE-D-26-07392

How Do Theme Park Attributes Influence Visitors' Positive Emotions? Nonlinear Effects and Spatial Heterogeneity in Shanghai's Theme Parks

The study analyzes theme parks in Shanghai, aiming to quantify visitors' positive emotions and identify associated attributes. To achieve this, it integrates online analytics, POI data and geospatial information, conduct spatial pattern analysis and develop interpretable predictive models. But perhaps these spatial analyzes have been little explored in the thematic figures presented and in the discussion of their efficiency.

I understand that the authors have made the corrections suggested in the first evaluation. And especially the analyses and discussions regarding the spatial approach in relation to the Park areas are now honest regarding the possibilities and many limitations in relation to the variables considered. I believe that the article can be ACCEPTED for publication in the journal.
