# Supplementary material for: How do theme park attributes influence visitors’ positive emotions? Nonlinear effects and spatial heterogeneity in Shanghai’s theme parks
Source: PLoS One. 2026 Jun 18;21(6):e0344051. doi: 10.1371/journal.pone.0344051 (PMC13278401; doi:10.1371/journal.pone.0344051)
Supplement: Attachment — Submitted filename: Response to Reviewers.docx [file pone.0344051.s005.docx]

**Point-to-point reply**

Dear Academic Editor and Reviewers

**PLoS ONE**

Manuscript Number: PONE-D-26-07392

Article Title: How Do Theme Park Attributes Influence Visitors’ Positive Emotions? Nonlinear Effects and Spatial Heterogeneity in Shanghai’s Theme Parks

We would like to thank you for your letter dated May 2, 2026. On behalf of all co-authors, we sincerely appreciate the opportunity to revise our manuscript. We are grateful for the constructive and insightful comments provided by the editor.

Our point-by-point responses to the comments from the editor and the two reviewers are attached to this letter. We have reproduced the comments below and provided our responses immediately afterward in a different color (blue).

Best regards,

# Xuehui Guo

**Response to the Academic Editor and Editorial Office**

Editor Requirement 1: Please ensure that your manuscript meets PLOS ONE’s style requirements, including those for file naming.

Response: Thank you for this reminder. We have carefully checked the revised manuscript against PLOS ONE’s formatting and file-submission requirements. In the resubmission package, we have prepared a marked-up revised manuscript, a clean manuscript, and a separate Response to Reviewers file, and we have aligned the manuscript structure, figure-caption presentation, and file organization with the journal’s instructions.

Editor Requirement 2: Please note that PLOS ONE has specific guidelines on code sharing for submissions in which author-generated code underpins the findings in the manuscript.

Response: We appreciate this requirement. Because the present study involves author-generated code for data processing, park-level dataset construction, modeling, and supplementary statistical testing, we have organized the relevant scripts used in the study and prepared them for sharing in a form that supports reproducibility and reuse, in line with the journal’s code-sharing policy.

Editor Requirement 3: Please confirm whether the submission contains all raw data required to replicate the results of your study. Authors must share the minimal data set for the submission.

Response: Thank you for this clarification. In response, we compiled the minimal dataset required to reproduce the reported findings, including the park-level analysis table, the values underlying the reported descriptive statistics, the values used to generate the figures, and the data required for the supplementary regression analyses. We have also revised the manuscript and supporting materials to ensure that the Data Availability Statement is consistent with the journal’s requirement for a minimal replicable dataset.

Editor Requirement 4: Please include a separate caption for each figure in your manuscript.

Response: We have checked the revised manuscript and ensured that each figure now has its own separate title and caption. We also revised the captions of the analytical figures so that their content, variables, and interpretive scope are more explicit and more closely aligned with the surrounding text.

Editor Requirement 5: Figures 1, 2, 4, and 5 in the original submission may contain copyrighted map images, which are not compatible with CC BY 4.0 publication unless permission is obtained.

Response: We fully appreciate this concern. To comply with the journal’s copyright policy, we did not retain proprietary basemap images. Instead, we prepared replacement figures using openly licensed or public-domain map data and revised the related figure captions accordingly. The revised versions of Figures 1, 2, 4, and 5 were therefore prepared specifically to avoid copyright incompatibility under the journal’s CC BY 4.0 requirements.

Editor Requirement 6: Figure 2 may contain copyrighted visual material and must either be licensed for CC BY 4.0 publication or replaced.

Response: We addressed this issue by redrawing Figure 2 as an author-generated replacement figure. The revised figure removes copyrighted visual elements and is intended to comply with the journal’s licensing requirements.

Editor Requirement 7: Please upload a new copy of Figure 2, as the detail is not clear.

Response: We have redrawn Figure 2 and improved its readability by enlarging the text in Step 3, simplifying the layout, and strengthening the visual hierarchy of the workflow. The revised figure is clearer and more legible than the original version.

Editor Requirement 8: If the reviewer comments include recommendations to cite specific previously published works, please evaluate whether those works are relevant and should be cited.

Response: We carefully re-examined the literature in light of the reviewers’ comments and updated the manuscript accordingly. In the revised version, we added relevant recent studies where appropriate, standardized citation formatting, and checked DOI and URL information to improve both the currency and the consistency of the references.

**General Response to the Academic Editor:**

Response: We are grateful to the Academic Editor and the Editorial Office for the detailed editorial guidance. In response, we revised the manuscript not only with respect to the reviewers’ scientific comments, but also with respect to the journal’s technical and submission requirements, including figure compliance, caption formatting, data availability, and code sharing. These revisions were made to improve the manuscript’s transparency, reproducibility, and compliance with PLOS ONE standards.

**Summary of the response:**

Response: This revision is not merely a minor polish but a systematic adjustment centered on five key aspects: “awareness of urban spatial issues—methodological transparency—formal statistical testing—conclusion boundaries—figure and data standards.”

• We strengthened the spatial problem orientation in the Introduction and Literature Review so that spatial analysis is motivated as part of the research question rather than presented merely as a technique.

• We expanded the Methods section, especially Section 3.1, to clarify the operational meaning of the park-level variables and added Section 3.5 to report the formal statistical testing strategy for nonlinear relationships.

• We added formal regression-based tests of functional form in Section 4.5, together with Table 8 and Figure 8, and revised the wording of the results accordingly. Only IP strength retained robust statistical support for nonlinearity; the nonlinear patterns of service quality and green-space leisure are now described as exploratory or unsupported.

• We reinterpreted the K-means results as attribute profiling groups rather than strong inferential park types, explicitly linked this to the low silhouette coefficient, and limited the ANOVA-based interpretation to exploratory comparison.

• We rewrote the Discussion, Recommendations, Limitations, and Conclusion sections to align them with the revised evidence, to avoid strong causal language, and to explain more clearly the value and limits of the spatial-analytical approach.

• We updated references, standardized formatting, revised figure captions, and prepared replacement versions of Figures 1, 2, 4, and 5 using open-source basemap data.

**Reviewer #1**

Reviewer #1: 1) Conduct formal statistical tests (e.g., piecewise regression or GAM) for the nonlinear effects (threshold, saturation) of key variables such as IP theme intensity and service quality, and report the test results. Do not rely solely on visual inspection of PDP plots.

Response: Thank you for highlighting that the original manuscript relied too heavily on visual inspection of the PDP plots when discussing threshold and saturation patterns. We fully agreed with this concern and therefore added formal statistical tests to verify the nonlinear patterns. In the original manuscript, Section 4.4 and Section 5.1 described IP theme intensity as showing a pronounced threshold effect and service quality as showing threshold-and-saturation characteristics without formal inferential support. In the revised manuscript, we added Section 3.5 (Formal Statistical Tests for Nonlinear Relationships), Section 4.5 (Regression Analysis), Table 8, and Figure 8. These additions introduce supplementary spline regression and segmented regression. Based on these tests, we have tightened the interpretation: only IP strength receives robust statistical support for nonlinearity, whereas the nonlinear patterns for service quality and green-space leisure are not statistically supported and are now described only as exploratory trends. The relevant revisions were made in Sections 3.5, 4.4, 4.5, 5.1, 5.3, and 5.4. We also report the key results directly in the text (for IP strength: spline F = 3.217, p = 0.027; segmented F = 10.975, p = 0.001; breakpoint ≈ 6.730). This revision directly addresses your request for formal statistical testing.

2) In the Methods section, describe in detail and transparently the quantification process for all core variables (IP intensity, service quality, green space leisure, etc.). This is critical for the replicability of the study.

Response: Thank you for emphasizing replicability and the need for transparent operationalization. We revised the Methods section substantially to make the park-level analysis framework explicit. In the original manuscript, Section 3.1 only stated that IP intensity, stimulation, green-space leisure, service quality, crowding, price, visitor flow, and accessibility had been collected, but it did not explain the role or meaning of each variable in the park-level model. In the revised manuscript, Section 3.1 now includes a dedicated paragraph that explains the meaning, unit, and scope of the key variables, including emotion_total, ticket_price, facility_count, distance_to_subway, service_population_15min, IP_strength, stimulation level, green-space leisure, service quality, crowding level, accessibility score, and park size category. We also clarify that the park-level analysis table serves as the modeling basis for the subsequent analyses. This change was made primarily in Section 3.1, with related consistency revisions in Sections 3.3 and 3.4 and in Table 1. In this way, the revised manuscript now presents not only the data sources but also the operational foundation of the park-level model more transparently.

3) Discuss the impact of the low K-means clustering quality (low silhouette score) on the subsequent ANOVA conclusions and avoid overinterpreting findings based on these groupings in the discussion.

Response: Thank you for this important caution. We agreed that the original manuscript risked overinterpreting the K-means groupings despite the low silhouette coefficient. In the original version, the low clustering quality (silhouette coefficient = 0.183) was noted, but the later discussion and recommendations still leaned too heavily on the three clusters as if they represented stable park types. In the revised manuscript, we have systematically downgraded the interpretive weight of these groups. Section 4.1 now explicitly states that, given the weak group separation, the ANOVA results are reported as exploratory descriptive evidence rather than validation of a stable typology. Section 5.2 further clarifies that the recommendations should be read as cross-sectoral priorities rather than rigid prescriptions for fixed park categories, and Section 5.3 lists the low silhouette coefficient as a study limitation. Across the manuscript, we now treat the K-means results as attribute profiling groups rather than strong latent types. These revisions directly address your concern about overinterpretation under low clustering quality.

4) In the Discussion section, delve deeper into the limitations imposed by the R² of 0.433 and suggest how future research might incorporate more variables to improve explanatory power.

Response: Thank you for asking us to discuss the boundary of the model’s explanatory power more explicitly. In the original manuscript, Section 4.4 briefly noted that approximately 43.3% of the variance was explained, but the implications of this result were not developed sufficiently in the Discussion. In the revised manuscript, Section 5.3 now explicitly interprets R² = 0.433 as meaningful but not exhaustive explanatory power. We also specify several omitted or insufficiently captured sources of variation, including weather, seasonality, special events, waiting time, promotional activities, companion composition, and sociodemographic differences. We then outline directions for future research, especially the integration of time-related, behavioral, and process-management variables. This revision creates a direct link between the model results in Section 4.4 and the limitations discussion in Section 5.3, thereby addressing your request in a more systematic way.

5) Ensure all citations are uniformly formatted and complete (e.g., check if reference [36], a Chinese policy document, meets the journal’s formatting requirements).

Response: Thank you for pointing out both the age profile and the formatting consistency of the references. We addressed both issues. In the original manuscript, many sources were dated before 2020, and the formatting of DOIs, URLs, policy documents, and software/platform references was inconsistent. In the revised manuscript, we added several newer studies in the Introduction, Literature Review, and Discussion, including recent work on tourism geographies, crowding, urban emotion, and spatial analytics. We also standardized the formatting of the references as far as possible, including DOI completion and normalized URL presentation. For policy documents and software/platform sources without DOI information, we adopted a consistent institutional-author–year–title–link format. These revisions were made throughout Sections 1, 2, 5.1, and the reference list, and they also respond to Reviewer #2’s concern that the literature was insufficiently up to date.

6) Figure captions should describe the content more clearly. For instance, the caption for Figure 7 could be revised to "Partial dependence plots illustrating the nonlinear effects of (a) IP theme intensity, (b) green space, and (c) service quality on predicted positive emotion scores."

Response: Thank you for this concrete suggestion. We revised the figure titles/captions and the corresponding in-text explanations to make the relationship between each figure and the related variables or models more explicit. In the original manuscript, the captioning was relatively brief, and much of the reader’s understanding depended on the order of subplots and the surrounding paragraph text. In the revised manuscript, the text in Sections 4.4 and 4.5 now explicitly links Figure 7 to the partial dependence relationships for IP strength, green-space leisure, and service quality, and links Figure 8 to the supplementary spline and segmented regression results. We also revised the wording of the figure titles/captions accordingly. In addition, because Figures 1, 2, 4, and 5 were redrawn using open-source map data and improved for clarity, the final submission package will include the updated figure files with the clearer captions. This revision directly addresses your suggestion regarding caption clarity and figure–text correspondence.

**Reviewer #2**

Reviewer #2: The study analyzes theme parks in Shanghai, aiming to quantify visitors’ positive emotions and identify associated attributes. To achieve this, it integrates online analytics, POI data, and geospatial information to conduct spatial pattern analysis and develop interpretable predictive models. However, these spatial analyses appear to have been insufficiently explored in the thematic figures presented and in the discussion of their effectiveness.

Therefore, we suggest some revisions to the text and figures presented.

Response: We thank the reviewer for pointing out the overall issue that “the spatial analysis thread is insufficient.” We agree that in the original manuscript, spatial methods appeared more as technical steps rather than being fully developed into a research question and interpretive framework. To address this, the revised manuscript systematically reinforces the Introduction, Methods, Results, and Discussion sections around the core motivation: “Why must we understand emotional differences in theme parks from an urban spatial scale?”

The original manuscript placed greater emphasis on “positive emotions themselves” in its research motivation, with spatial analysis appearing primarily in the Methods and Results sections. The revised manuscript explicitly states in “1. Introduction” that theme parks are not uniformly distributed but are embedded within distinct spatial supply structures. Accessibility, agglomeration, location, and the surrounding recreational environment alter visitors’ conditions of visit and experiential expectations; therefore, discussions of positive emotions cannot be separated from the urban spatial scale.In “4.3 Spatial Pattern Analysis,” Figures 4 and 5 are explicitly linked to “where clusters form” and “whether emotions within clusters are consistent,” respectively.In “5.1 Discussion” and “5.3 Limitations and Future Directions,” the roles and limitations of the methodological combination of ANN, KDE, and emotional overlay maps have been further elaborated. The main revisions are located in “1 Introduction,” “4.3 Spatial Pattern Analysis,” “5.1 Discussion,” “5.3 Limitations and Future Directions,” and “5.4 Conclusion.”

• Sections 1 (Introduction) and 2 (Theoretical Foundations and Literature Review) do not reflect the spatial methodological approach described in the Methodology section and presented in the Results. The emphasis is on “positive emotions” related to tourism, with no mention of the spatial approach. It is only through summarizing the ideas presented—without references—that the text addresses this: “In summary, this study employs Shanghai as its empirical setting, integrating text sentiment quantification, spatial pattern analysis, and multivariate modeling to deeply analyze the influence mechanisms and differential effects of various theme park attributes on visitors’ positive emotions.”;

Response: We thank the reviewer for raising this critical point. We have restructured the introduction and literature review to highlight the central thread of spatial research, ensuring that spatial analysis is no longer merely a methodological introduction but an integral part of the research rationale.

The original introduction primarily emphasized the importance of theme parks, the significance of positive emotions, and the potential of big data, but lacked the research question of “why it is necessary to examine this from an urban spatial scale.” The revised version restructures “1. Introduction” into four paragraphs: The first paragraph argues that research on emotions in megacity theme parks cannot be separated from the urban spatial scale.The second paragraph explains the suitability of Shanghai as a case study. The third paragraph summarizes research gaps regarding the urban scale, multi-source data, and spatial awareness. The fourth paragraph concludes the text with three research questions and explicitly positions spatial analysis as a tool to explain “why similar parks exhibit different emotional outcomes in different locations.”At the same time, the discussion on “how emotions vary with spatial touchpoints and service processes” has been retained and strengthened in “2. Theoretical Framework and Literature Review,” creating a clearer link between the spatial dimension and attribution mechanisms. The main revisions are found in paragraphs 1–4 of “1. Introduction” and the first half of “2. Theoretical Framework and Literature Review.”

• How do theme park attributes influence visitors' positive emotions? or What is the influence of theme park attributes on visitors’ positive emotions?

Response: Thank you for the reviewer’s suggestion regarding the phrasing of the title and research questions. We have now aligned the wording across the abstract, research questions, and conclusions.

Although the original manuscript used the research-oriented title “How Do Theme Park Attributes Influence Visitors’ Positive Emotions?”, the text within the introduction occasionally shifted toward “studying positive emotions,” which was not entirely aligned with the focus of the title. The revised manuscript consistently focuses on “how theme park attributes influence visitors’ positive emotions and how this influence varies at the urban spatial scale” in the abstract, introduction, and conclusion.Additionally, the fourth paragraph clearly lists three research questions, corresponding to the importance of attributes, the nature of relationships, and spatial clustering/heterogeneity. Therefore, the main revisions are concentrated in the “Abstract,” the fourth paragraph of “1. Introduction,” and the “5.4 Conclusions” section. The title has not been changed.

• review the structure of the text; the paragraphs are very long and contain some repeated questions. For example, there are paragraphs in the Results section that practically take up an entire page;;

Response: We appreciate the reviewer’s comments regarding text structure and paragraph length. We have split and reorganized the lengthy paragraphs in the Introduction, Results, and Discussion sections to reduce repetition and enhance clarity.

In the original manuscript, the points “the importance of theme parks,” “the importance of positive emotions,” “the potential of online reviews,” and “the lack of city-scale studies” were repeatedly emphasized between paragraphs 2 and 4 of the Introduction. Similarly, the Results and Discussion sections contained paragraphs overloaded with information. The revised manuscript condenses the Introduction into a four-paragraph structure, avoiding the repetitive emphasis on “the importance of positive emotions.”In the Results section, the analysis of Random Forest and formal regression tests has been separated into “4.4 Analysis of Random Forest Model Results” and the newly added “4.5 Regression Analysis.” In the Discussion, IP theme intensity, service quality, green_space_leisure, scale/accessibility, and reflections on spatial methods have been expanded into separate sections. The main revisions are located in “1 Introduction,” “4.4,” “4.5,” and “5.1–5.3.”

• in Table 2 in Section 3.3, I suggest standardizing GS or GSR in relation to green spaces;

Response: Thank you for noticing this terminology inconsistency. We standardized the wording in Table 2 and the corresponding text. In the original manuscript, the table and related descriptions used inconsistent labels such as GS or GSR. In the revised manuscript, we use the unified term 'green-space leisure' in Table 2, its note, and the surrounding text in Section 3.3. This correction improves consistency across the Methods, Results, and Discussion sections.

• I suggest that the text in section 3.3, “The average contour coefficient for clustering was 0.183, indicating relatively blurred group boundaries,” be presented in the Results section rather than the Methods section;

Response: We appreciate the reviewer’s suggestion. We have moved the “interpretive implications” of the contour coefficient primarily to the Results and Discussion sections, while retaining only the most essential explanations in the Methods section.

Given that K-means was used in this study to form attribute profile groups, the revised manuscript retains a necessary explanation regarding the contour coefficient in “3.3 Spatial Analysis and Park Classification” to clarify why the clustering results are not considered strong inferential statistics. Additionally, in “4.1 Descriptive Statistics and Park Classification,” we explicitly state that since the contour coefficient is only 0.183, ANOVA is used solely for exploratory description.This limitation is further discussed in Section 5.3, “Research Limitations and Future Directions.” In other words, we have partially accepted this suggestion: the Methods section retains the most basic quality notes, while the interpretive implications of the contour coefficient are primarily shifted to the Results and Limitations sections. The main revisions are located in Sections 3.3, 4.1, and 5.3.

• In general, references have not been updated. Only 10 of the 48 references are from after 2020;

Response: Thank you. We addressed this issue by updating the literature base and standardizing the references. In the revised manuscript, we incorporated more recent studies in the Introduction, Literature Review, and Discussion, including recent work on tourism geographies, crowding research, urban emotion, and image/social-media-based analytics. We also revised the formatting of the reference list to improve consistency. This change responds both to your concern about literature currency and to Reviewer #1’s comment on reference completeness and formatting.

• I suggest increasing the text font in Figure 2 (Step 3 multivariate model);

Response: Thank you for the reviewer’s specific suggestion regarding the clarity of Figure 2. We have redrawn Figure 2, enlarging the font in the Step 3 section, simplifying visual elements, and improving overall readability in the revised version.

In the original Figure 2, the font size for the “multivariate model” section in Step 3 was too small, and the elements within the figure were densely packed. Based on feedback from the editor and reviewers, we have redrawn the figure, addressing both copyright and clarity issues. This primarily concerns the final submitted version of “Figure 2: Research Flowchart.” The description of the research process in the main text of the revised manuscript has also been updated to align with the new Figure 2.

• Figures 1, 4, and 5 do not indicate the locations of the Pudong New Area and Songjiang District. I suggest including these locations on the map, as they are key areas of spatial concentration for tourism activities and will help readers follow the authors’ analyses;

Response: Thank you for this practical recommendation. We accepted it and revised the map figures accordingly. In the original manuscript, the text referred to Pudong New Area and Songjiang District, but the figures themselves did not mark these locations clearly. The revised map has been redrawn using open-license map data, and Songjiang District has been clearly labeled in this version to enable readers to more intuitively follow the analysis in Section 4.3. Since the density of theme parks in Songjiang District is higher than that in Pudong District, the labeling and analysis of Pudong District have been omitted. This improves the correspondence between the map and the spatial discussion.

• I have the impression that the analysis of Figures 4 and 5 is not consistent with the text presented and that the order of these figures is incorrect;

Response: Thank you. We carefully rechecked the division of labor, ordering, and textual interpretation of Figures 4 and 5. In the revised manuscript, Section 4.3 first presents the ANN and KDE results and then interprets Figure 4 specifically as the kernel density map showing where parks cluster and where hotspot structures are located. Figure 5 is then used to discuss whether emotional heterogeneity persists within those clustered areas. This revision aligns the figure order with the analytic sequence and clarifies the distinct function of each figure.

• It seems that Figure 4 does not allow the map to read, as described in item 4.3, the spatial relationships between park sizes and the average of positive emotions;

Response: Thank you for this observation. We tightened the functional definition of Figure 4 in the revised manuscript. Rather than implying that Figure 4 simultaneously represents density structure and the relationship between park size and average positive emotion, we now use Figure 4 strictly for hotspot identification via kernel density estimation. The discussion of emotional differences within clustered areas is now explicitly assigned to Figure 5. In this way, each figure corresponds to what it can actually show.

• Apparently, Figure 6 shows how little influence “area” has on “Importance contributions”;

Response: Thank you for this helpful caution. We revised the text to avoid implying that park size or area is one of the core drivers in the feature-importance analysis. In the revised manuscript, Figure 6 is interpreted only in terms of the variables that actually rank highly in the random forest analysis, such as IP strength, service quality, and green-space leisure. Park size remains in Section 4.1 as an external grouping variable for exploratory comparison, but it is no longer suggested to be a dominant contributor in the feature-importance analysis. This change separates the size-group comparison from the model-based feature ranking more clearly.

• Figure 1 shows, in Shanghai, the parks and the road network (perhaps in terms of accessibility, but a topic little covered in the analysis). However, no map presents green spaces, a relevant topic in the analysis of section 4.4 of the Results and at the beginning of the Discussion and Conclusions;

Response: Thank you. We considered this point carefully and clarified the relationship between variable definition and figure function. In the revised manuscript, we make clear in Section 3.1 that green-space leisure is a park-level composite indicator rather than a direct mapped layer equivalent to citywide green-cover distribution. For this reason, we did not add a separate green-space base map that might visually imply a different operational meaning from the one used in the model. Instead, we revised Sections 4.4, 4.5, and 5.1 to explain the statistical meaning and role of green-space leisure more accurately and to keep the maps focused on park distribution, accessibility context, and hotspot structure. In other words, rather than adding a potentially misleading map layer, we clarified the scope and meaning of the variable itself.

• I highlight that nothing in the Discussions, Recommendations and Conclusions was addressed regarding the approach and efficiency of the methodology used in spatial analysis.

Response: Thank you for emphasizing this gap. We addressed it by adding a methodological reflection on the role and limits of the spatial analysis in the Discussion, Recommendations, Limitations, and Conclusion sections. In the revised manuscript, Section 5.1 now explains that the spatial methods answer three linked questions: whether clustering exists, where hotspot structures are located, and whether emotional heterogeneity persists within those hotspot areas. Section 5.2 then translates this into a planning implication—namely, that urban governance should move beyond simple hotspot logic. Section 5.3 explicitly states that spatial analysis can identify clustering and hotspot heterogeneity but cannot by itself establish spatial spillover effects or causality. Section 5.4 retains the same cautious position in the conclusion. This set of revisions was made specifically to bring the efficiency, usefulness, and boundary of the spatial-analytical approach into the manuscript’s interpretive core.

Dear Editor,

We sincerely appreciate the editor’s and reviewers’ constructive comments. We believe that the manuscript has been substantially improved in terms of methodological transparency, evidential caution, spatial problem orientation, figure clarity, and overall academic rigor. We hope that the revised version now addresses the concerns raised during review, and we would be grateful for the opportunity to have the manuscript considered further for publication in PLOS ONE.

Best regards,

Xuehui Guo
