# Supplementary material for: How do theme park attributes influence visitors’ positive emotions? Nonlinear effects and spatial heterogeneity in Shanghai’s theme parks
Source: PLoS One. 2026 Jun 18;21(6):e0344051. doi: 10.1371/journal.pone.0344051 (PMC13278401; doi:10.1371/journal.pone.0344051)
Supplement: Peer review history

# Peer review: Decision Letter 0

**DOI:** 10.1371/journal.pone.0344051.r001

**Authors:** Renwu Wu

18 Mar 2026

PONE-D-26-07392

How Do Theme Park Attributes Influence Visitors’ Positive Emotions? Nonlinear Effects and Spatial Heterogeneity in Shanghai’s Theme Parks

PLOS One

Dear Dr. Gao,

Thank you for submitting your manuscript to PLOS ONE. After careful consideration, we feel that it has merit but does not fully meet PLOS ONE’s publication criteria as it currently stands. Therefore, we invite you to submit a revised version of the manuscript that addresses the points raised during the review process.

Please submit your revised manuscript by May 02 2026 11:59PM. If you will need more time than this to complete your revisions, please reply to this message or contact the journal office at plosone@plos.org. When you're ready to submit your revision, log on to https://www.editorialmanager.com/pone/ and select the 'Submissions Needing Revision' folder to locate your manuscript file.

Please include the following items when submitting your revised manuscript:

- A letter that responds to each point raised by the academic editor and reviewer(s). You should upload this letter as a separate file labeled 'Response to Reviewers'.
- A marked-up copy of your manuscript that highlights changes made to the original version. You should upload this as a separate file labeled 'Revised Manuscript with Track Changes'.
- An unmarked version of your revised paper without tracked changes. You should upload this as a separate file labeled 'Manuscript'.

If you would like to make changes to your financial disclosure, please include your updated statement in your cover letter. Guidelines for resubmitting your figure files are available below the reviewer comments at the end of this letter.

If applicable, we recommend that you deposit your laboratory protocols in protocols.io to enhance the reproducibility of your results. Protocols.io assigns your protocol its own identifier (DOI) so that it can be cited independently in the future. For instructions see: https://journals.plos.org/plosone/s/submission-guidelines#loc-laboratory-protocols. Additionally, PLOS ONE offers an option for publishing peer-reviewed Lab Protocol articles, which describe protocols hosted on protocols.io. Read more information on sharing protocols at https://plos.org/protocols?utm_medium=editorial-email&utm_source=authorletters&utm_campaign=protocols.

We look forward to receiving your revised manuscript.

Kind regards,

Renwu Wu

Academic Editor

PLOS One

**Journal Requirements:**

1. When submitting your revision, we need you to address these additional requirements.

Please ensure that your manuscript meets PLOS ONE's style requirements, including those for file naming. The PLOS ONE style templates can be found at

https://journals.plos.org/plosone/s/file?id=wjVg/PLOSOne_formatting_sample_main_body.pdf and

https://journals.plos.org/plosone/s/file?id=ba62/PLOSOne_formatting_sample_title_authors_affiliations.pdf

2. Please note that PLOS One has specific guidelines on code sharing for submissions in which author-generated code underpins the findings in the manuscript. In these cases, we expect all author-generated code to be made available without restrictions upon publication of the work. Please review our guidelines at https://journals.plos.org/plosone/s/materials-and-software-sharing#loc-sharing-code and ensure that your code is shared in a way that follows best practice and facilitates reproducibility and reuse.

3. We note that your Data Availability Statement is currently as follows: All relevant data are within the manuscript and its Supporting Information files.

Please confirm at this time whether or not your submission contains all raw data required to replicate the results of your study. Authors must share the “minimal data set” for their submission. PLOS defines the minimal data set to consist of the data required to replicate all study findings reported in the article, as well as related metadata and methods (https://journals.plos.org/plosone/s/data-availability#loc-minimal-data-set-definition).

For example, authors should submit the following data:

- The values behind the means, standard deviations and other measures reported;

- The values used to build graphs;

- The points extracted from images for analysis.

Authors do not need to submit their entire data set if only a portion of the data was used in the reported study.

If your submission does not contain these data, please either upload them as Supporting Information files or deposit them to a stable, public repository and provide us with the relevant URLs, DOIs, or accession numbers. For a list of recommended repositories, please see https://journals.plos.org/plosone/s/recommended-repositories.

If there are ethical or legal restrictions on sharing a de-identified data set, please explain them in detail (e.g., data contain potentially sensitive information, data are owned by a third-party organization, etc.) and who has imposed them (e.g., an ethics committee). Please also provide contact information for a data access committee, ethics committee, or other institutional body to which data requests may be sent. If data are owned by a third party, please indicate how others may request data access.

4. Please include a separate caption for each figure in your manuscript.

5. We note that Figures 1, 2, 4 and 5 in your submission contain map images which may be copyrighted. All PLOS content is published under the Creative Commons Attribution License (CC BY 4.0), which means that the manuscript, images, and Supporting Information files will be freely available online, and any third party is permitted to access, download, copy, distribute, and use these materials in any way, even commercially, with proper attribution. For these reasons, we cannot publish previously copyrighted maps or satellite images created using proprietary data, such as Google software (Google Maps, Street View, and Earth). For more information, see our copyright guidelines: http://journals.plos.org/plosone/s/licenses-and-copyright.

We require you to either present written permission from the copyright holder to publish these figures specifically under the CC BY 4.0 license, or remove the figures from your submission:

a. You may seek permission from the original copyright holder of Figures 1, 2, 4 and 5 to publish the content specifically under the CC BY 4.0 license.

We recommend that you contact the original copyright holder with the Content Permission Form (http://journals.plos.org/plosone/s/file?id=7c09/content-permission-form.pdf) and the following text:

“I request permission for the open-access journal PLOS ONE to publish XXX under the Creative Commons Attribution License (CCAL) CC BY 4.0 (http://creativecommons.org/licenses/by/4.0/). Please be aware that this license allows unrestricted use and distribution, even commercially, by third parties. Please reply and provide explicit written permission to publish XXX under a CC BY license and complete the attached form.”

Please upload the completed Content Permission Form or other proof of granted permissions as an "Other" file with your submission.

In the figure caption of the copyrighted figure, please include the following text: “Reprinted from [ref] under a CC BY license, with permission from [name of publisher], original copyright [original copyright year].”

b. If you are unable to obtain permission from the original copyright holder to publish these figures under the CC BY 4.0 license or if the copyright holder’s requirements are incompatible with the CC BY 4.0 license, please either i) remove the figure or ii) supply a replacement figure that complies with the CC BY 4.0 license. Please check copyright information on all replacement figures and update the figure caption with source information. If applicable, please specify in the figure caption text when a figure is similar but not identical to the original image and is therefore for illustrative purposes only.

The following resources for replacing copyrighted map figures may be helpful:

USGS National Map Viewer (public domain): http://viewer.nationalmap.gov/viewer/

The Gateway to Astronaut Photography of Earth (public domain): http://eol.jsc.nasa.gov/sseop/clickmap/

Maps at the CIA (public domain): https://www.cia.gov/library/publications/the-world-factbook/index.html and https://www.cia.gov/library/publications/cia-maps-publications/index.html

NASA Earth Observatory (public domain): http://earthobservatory.nasa.gov/

Landsat: http://landsat.visibleearth.nasa.gov/

USGS EROS (Earth Resources Observatory and Science (EROS) Center) (public domain): http://eros.usgs.gov/#

Natural Earth (public domain): http://www.naturalearthdata.com/

6. We note that Figure 2 in your submission contain copyrighted images. All PLOS content is published under the Creative Commons Attribution License (CC BY 4.0), which means that the manuscript, images, and Supporting Information files will be freely available online, and any third party is permitted to access, download, copy, distribute, and use these materials in any way, even commercially, with proper attribution. For more information, see our copyright guidelines: http://journals.plos.org/plosone/s/licenses-and-copyright.

We require you to either present written permission from the copyright holder to publish these figures specifically under the CC BY 4.0 license, or remove the figures from your submission:

a. You may seek permission from the original copyright holder of Figure 2 to publish the content specifically under the CC BY 4.0 license.

We recommend that you contact the original copyright holder with the Content Permission Form (http://journals.plos.org/plosone/s/file?id=7c09/content-permission-form.pdf) and the following text:

“I request permission for the open-access journal PLOS ONE to publish XXX under the Creative Commons Attribution License (CCAL) CC BY 4.0 (http://creativecommons.org/licenses/by/4.0/). Please be aware that this license allows unrestricted use and distribution, even commercially, by third parties. Please reply and provide explicit written permission to publish XXX under a CC BY license and complete the attached form.”

Please upload the completed Content Permission Form or other proof of granted permissions as an "Other" file with your submission.

In the figure caption of the copyrighted figure, please include the following text: “Reprinted from [ref] under a CC BY license, with permission from [name of publisher], original copyright [original copyright year].”

b. If you are unable to obtain permission from the original copyright holder to publish these figures under the CC BY 4.0 license or if the copyright holder’s requirements are incompatible with the CC BY 4.0 license, please either i) remove the figure or ii) supply a replacement figure that complies with the CC BY 4.0 license. Please check copyright information on all replacement figures and update the figure caption with source information. If applicable, please specify in the figure caption text when a figure is similar but not identical to the original image and is therefore for illustrative purposes only.

7. Please upload a new copy of Figure 2, as the detail is not clear. Please follow the link for more information:  https://journals.plos.org/plosone/s/figures

8. If the reviewer comments include a recommendation to cite specific previously published works, please review and evaluate these publications to determine whether they are relevant and should be cited. There is no requirement to cite these works unless the editor has indicated otherwise.

[Note: HTML markup is below. Please do not edit.]

Reviewers' comments:

Reviewer's Responses to Questions

**Comments to the Author**

1. Is the manuscript technically sound, and do the data support the conclusions?

The manuscript must describe a technically sound piece of scientific research with data that supports the conclusions. Experiments must have been conducted rigorously, with appropriate controls, replication, and sample sizes. The conclusions must be drawn appropriately based on the data presented.

Reviewer #1: Partly

Reviewer #2: Partly

**********

2. Has the statistical analysis been performed appropriately and rigorously?

Reviewer #1: N/A

Reviewer #2: Yes

**********

3. Have the authors made all data underlying the findings in their manuscript fully available?

The PLOS Data policy requires authors to make all data underlying the findings described in their manuscript fully available without restriction, with rare exception (please refer to the Data Availability Statement in the manuscript PDF file). The data should be provided as part of the manuscript or its supporting information, or deposited to a public repository. For example, in addition to summary statistics, the data points behind means, medians and variance measures should be available. If there are restrictions on publicly sharing data—e.g. participant privacy or use of data from a third party—those must be specified.

Reviewer #1: No

Reviewer #2: Yes

**********

4. Is the manuscript presented in an intelligible fashion and written in standard English?

PLOS ONE does not copyedit accepted manuscripts, so the language in submitted articles must be clear, correct, and unambiguous. Any typographical or grammatical errors should be corrected at revision, so please note any specific errors here.

Reviewer #1: Yes

Reviewer #2: Yes

**********

5. Review Comments to the Author

Please use the space provided to explain your answers to the questions above. You may also include additional comments for the author, including concerns about dual publication, research ethics, or publication ethics. (Please upload your review as an attachment if it exceeds 20,000 characters)

**Reviewer #1:**   1）Conduct formal statistical tests (e.g., piecewise regression or GAM) for the nonlinear effects (threshold, saturation) of key variables like IP theme intensity and service quality, and report the test results. Do not rely solely on visual inspection of PDP plots.

2）In the Methods section, describe in detail and transparently the quantification process for all core variables (IP intensity, service quality, green space leisure, etc.). This is critical for the replicability of the study.

3）Discuss the impact of the low K-means clustering quality (low silhouette score) on the subsequent ANOVA conclusions and avoid overinterpreting findings based on these groupings in the discussion.

4）In the Discussion section, delve deeper into the limitations imposed by the R² of 0.433 and suggest how future research might incorporate more variables to improve explanatory power.

5）Ensure all citations are uniformly formatted and complete (e.g., check if reference [36], a Chinese policy document, meets the journal's formatting requirements).

6）Figure captions should describe the content more clearly. For instance, the caption for Figure 7 could be revised to "Partial dependence plots illustrating the nonlinear effects of (a) IP theme intensity, (b) green space, and (c) service quality on predicted positive emotion scores."

**Reviewer #2:**  The study analyzes theme parks in Shanghai, aiming to quantify visitors' positive emotions and identify associated attributes. To achieve this, it integrates online analytics, POI data and geospatial information, conduct spatial pattern analysis and develop interpretable predictive models. But perhaps these spatial analyzes have been little explored in the thematic figures presented and in the discussion of their efficiency.

Therefore, we suggest some revisions to the text and figures presented.

Comments

• Hi topics 1 (Introduction) and 2 (Theoretical Foundations and Literature Review) do not reflect the spatial methodological approach described in the Methodology and presented in the Results. The emphasis is on "positive emotions" related to tourism and nothing on the spatial approach. It is practically by summarizing the ideas presented that, without references, addresses that: “In summary, this study employs Shanghai as its empirical setting, integrating text sentiment quantification, spatial pattern analysis, and multivariate modelling to deeply analyse the influence mechanisms and differential effects of various theme park attributes on visitors' positive emotions.“;

• How do theme park attributes influence visitors' positive emotions? or What is the influence of theme park attributes on visitors’ positive emotions?

• review the structure of the text, the paragraphs are very long and contain some repeated questions. For example, there are paragraphs in the Results that practically take up an entire page;;

• in Table 2 in item 3.3, I suggest standardizing GS or GSR in relation to green spaces;

• I suggest that the text in item 3.3 “The average contour coefficient for clustering was 0.183, indicating relatively blurred group boundaries.” be presented in results and not in methodology;

• In general, references are not updated. Only 10 of the 48 references are from after 2020;

• I suggest increasing the text font in Figure 2 (Step 3 multivariate model);

• Figures 1, 4 and 5 make no mention of the location of the Pudong New Area and Songjiang District. I suggest including these locations on the map, as they are important areas of spatial concentration of tourism activities and will allow you to follow the authors' analyses;

• I have the impression that the analysis of Figures 4 and 5 is not compatible with the text presented and that the order of these figures is wrong;

• It seems that Figure 4 does not allow the map to read, as described in item 4.3, the spatial relationships between park sizes and the average of positive emotions;

• Apparently Figure 6 shows how little influence the "area" has on "Importance contributions";

• Figure 1 shows, in Shanghai, the parks and the road network (perhaps in terms of accessibility, but a topic little covered in the analysis). However, no map presents green spaces, a relevant topic in the analysis of topic 4.4 of the Results and at the beginning of the Discussion and Conclusions;

• I highlight that nothing in the Discussions, Recommendations and Conclusions was addressed regarding the approach and efficiency of the methodology used in spatial analysis.

**********

6. PLOS authors have the option to publish the peer review history of their article (what does this mean?). If published, this will include your full peer review and any attached files.

If you choose “no”, your identity will remain anonymous but your review may still be made public.

**Do you want your identity to be public for this peer review?**   For information about this choice, including consent withdrawal, please see our Privacy Policy.

Reviewer #1: No

Reviewer #2: No

**********

[NOTE: If reviewer comments were submitted as an attachment file, they will be attached to this email and accessible via the submission site. Please log into your account, locate the manuscript record, and check for the action link "View Attachments". If this link does not appear, there are no attachment files.]

To ensure your figures meet our technical requirements, please review our figure guidelines: https://journals.plos.org/plosone/s/figures

You may also use PLOS’s free figure tool, NAAS, to help you prepare publication quality figures: https://journals.plos.org/plosone/s/figures#loc-tools-for-figure-preparation.

NAAS will assess whether your figures meet our technical requirements by comparing each figure against our figure specifications.

Attachment Submitted filename: REVIEWER PONE-D-26-07392.pdf

# Author comment: Author response to Decision Letter 1

**DOI:** 10.1371/journal.pone.0344051.r002

5 May 2026

Dear Academic Editor and Reviewers

PLoS ONE

Manuscript Number: PONE-D-26-07392

Article Title: How Do Theme Park Attributes Influence Visitors’ Positive Emotions? Nonlinear Effects and Spatial Heterogeneity in Shanghai’s Theme Parks

We would like to thank you for your letter dated May 2, 2026. On behalf of all co-authors, we sincerely appreciate the opportunity to revise our manuscript. We are grateful for the constructive and insightful comments provided by the editor.

Our point-by-point responses to the comments from the editor and the two reviewers are attached to this letter. We have reproduced the comments below and provided our responses immediately afterward in a different color (blue).

Best regards,

Xuehui Guo

Response to the Academic Editor and Editorial Office

Editor Requirement 1: Please ensure that your manuscript meets PLOS ONE’s style requirements, including those for file naming.

Response: Thank you for this reminder. We have carefully checked the revised manuscript against PLOS ONE’s formatting and file-submission requirements. In the resubmission package, we have prepared a marked-up revised manuscript, a clean manuscript, and a separate Response to Reviewers file, and we have aligned the manuscript structure, figure-caption presentation, and file organization with the journal’s instructions.

Editor Requirement 2: Please note that PLOS ONE has specific guidelines on code sharing for submissions in which author-generated code underpins the findings in the manuscript.

Response: We appreciate this requirement. Because the present study involves author-generated code for data processing, park-level dataset construction, modeling, and supplementary statistical testing, we have organized the relevant scripts used in the study and prepared them for sharing in a form that supports reproducibility and reuse, in line with the journal’s code-sharing policy.

Editor Requirement 3: Please confirm whether the submission contains all raw data required to replicate the results of your study. Authors must share the minimal data set for the submission.

Response: Thank you for this clarification. In response, we compiled the minimal dataset required to reproduce the reported findings, including the park-level analysis table, the values underlying the reported descriptive statistics, the values used to generate the figures, and the data required for the supplementary regression analyses. We have also revised the manuscript and supporting materials to ensure that the Data Availability Statement is consistent with the journal’s requirement for a minimal replicable dataset.

Editor Requirement 4: Please include a separate caption for each figure in your manuscript.

Response: We have checked the revised manuscript and ensured that each figure now has its own separate title and caption. We also revised the captions of the analytical figures so that their content, variables, and interpretive scope are more explicit and more closely aligned with the surrounding text.

Editor Requirement 5: Figures 1, 2, 4, and 5 in the original submission may contain copyrighted map images, which are not compatible with CC BY 4.0 publication unless permission is obtained.

Response: We fully appreciate this concern. To comply with the journal’s copyright policy, we did not retain proprietary basemap images. Instead, we prepared replacement figures using openly licensed or public-domain map data and revised the related figure captions accordingly. The revised versions of Figures 1, 2, 4, and 5 were therefore prepared specifically to avoid copyright incompatibility under the journal’s CC BY 4.0 requirements.

Editor Requirement 6: Figure 2 may contain copyrighted visual material and must either be licensed for CC BY 4.0 publication or replaced.

Response: We addressed this issue by redrawing Figure 2 as an author-generated replacement figure. The revised figure removes copyrighted visual elements and is intended to comply with the journal’s licensing requirements.

Editor Requirement 7: Please upload a new copy of Figure 2, as the detail is not clear.

Response: We have redrawn Figure 2 and improved its readability by enlarging the text in Step 3, simplifying the layout, and strengthening the visual hierarchy of the workflow. The revised figure is clearer and more legible than the original version.

Editor Requirement 8: If the reviewer comments include recommendations to cite specific previously published works, please evaluate whether those works are relevant and should be cited.

Response: We carefully re-examined the literature in light of the reviewers’ comments and updated the manuscript accordingly. In the revised version, we added relevant recent studies where appropriate, standardized citation formatting, and checked DOI and URL information to improve both the currency and the consistency of the references.

General Response to the Academic Editor:

Response: We are grateful to the Academic Editor and the Editorial Office for the detailed editorial guidance. In response, we revised the manuscript not only with respect to the reviewers’ scientific comments, but also with respect to the journal’s technical and submission requirements, including figure compliance, caption formatting, data availability, and code sharing. These revisions were made to improve the manuscript’s transparency, reproducibility, and compliance with PLOS ONE standards.

Summary of the response:

Response: This revision is not merely a minor polish but a systematic adjustment centered on five key aspects: “awareness of urban spatial issues—methodological transparency—formal statistical testing—conclusion boundaries—figure and data standards.”

• We strengthened the spatial problem orientation in the Introduction and Literature Review so that spatial analysis is motivated as part of the research question rather than presented merely as a technique.

• We expanded the Methods section, especially Section 3.1, to clarify the operational meaning of the park-level variables and added Section 3.5 to report the formal statistical testing strategy for nonlinear relationships.

• We added formal regression-based tests of functional form in Section 4.5, together with Table 8 and Figure 8, and revised the wording of the results accordingly. Only IP strength retained robust statistical support for nonlinearity; the nonlinear patterns of service quality and green-space leisure are now described as exploratory or unsupported.

• We reinterpreted the K-means results as attribute profiling groups rather than strong inferential park types, explicitly linked this to the low silhouette coefficient, and limited the ANOVA-based interpretation to exploratory comparison.

• We rewrote the Discussion, Recommendations, Limitations, and Conclusion sections to align them with the revised evidence, to avoid strong causal language, and to explain more clearly the value and limits of the spatial-analytical approach.

• We updated references, standardized formatting, revised figure captions, and prepared replacement versions of Figures 1, 2, 4, and 5 using open-source basemap data.

Reviewer #1

Reviewer #1: 1) Conduct formal statistical tests (e.g., piecewise regression or GAM) for the nonlinear effects (threshold, saturation) of key variables such as IP theme intensity and service quality, and report the test results. Do not rely solely on visual inspection of PDP plots.

Response: Thank you for highlighting that the original manuscript relied too heavily on visual inspection of the PDP plots when discussing threshold and saturation patterns. We fully agreed with this concern and therefore added formal statistical tests to verify the nonlinear patterns. In the original manuscript, Section 4.4 and Section 5.1 described IP theme intensity as showing a pronounced threshold effect and service quality as showing threshold-and-saturation characteristics without formal inferential support. In the revised manuscript, we added Section 3.5 (Formal Statistical Tests for Nonlinear Relationships), Section 4.5 (Regression Analysis), Table 8, and Figure 8. These additions introduce supplementary spline regression and segmented regression. Based on these tests, we have tightened the interpretation: only IP strength receives robust statistical support for nonlinearity, whereas the nonlinear patterns for service quality and green-space leisure are not statistically supported and are now described only as exploratory trends. The relevant revisions were made in Sections 3.5, 4.4, 4.5, 5.1, 5.3, and 5.4. We also report the key results directly in the text (for IP strength: spline F = 3.217, p = 0.027; segmented F = 10.975, p = 0.001; breakpoint ≈ 6.730). This revision directly addresses your request for formal statistical testing.

2) In the Methods section, describe in detail and transparently the quantification process for all core variables (IP intensity, service quality, green space leisure, etc.). This is critical for the replicability of the study.

Response: Thank you for emphasizing replicability and the need for transparent operationalization. We revised the Methods section substantially to make the park-level analysis framework explicit. In the original manuscript, Section 3.1 only stated that IP intensity, stimulation, green-space leisure, service quality, crowding, price, visitor flow, and accessibility had been collected, but it did not explain the role or meaning of each variable in the park-level model. In the revised manuscript, Section 3.1 now includes a dedicated paragraph that explains the meaning, unit, and scope of the key variables, including emotion_total, ticket_price, facility_count, distance_to_subway, service_population_15min, IP_strength, stimulation level, green-space leisure, service quality, crowding level, accessibility score, and park size category. We also clarify that the park-level analysis table serves as the modeling basis for the subsequent analyses. This change was made primarily in Section 3.1, with related consistency revisions in Sections 3.3 and 3.4 and in Table 1. In this way, the revised manuscript now presents not only the data sources but also the operational foundation of the park-level model more transparently.

3) Discuss the impact of the low K-means clustering quality (low silhouette score) on the subsequent ANOVA conclusions and avoid overinterpreting findings based on these groupings in the discussion.

Response: Thank you for this important caution. We agreed that the original manuscript risked overinterpreting the K-means groupings despite the low silhouette coefficient. In the original version, the low clustering quality (silhouette coefficient = 0.183) was noted, but the later discussion and recommendations still leaned too heavily on the three clusters as if they represented stable park types. In the revised manuscript, we have systematically downgraded the interpretive weight of these groups. Section 4.1 now explicitly states that, given the weak group separation, the ANOVA results are reported as exploratory descriptive evidence rather than validation of a stable typology. Section 5.2 further clarifies that the recommendations should be read as cross-sectoral priorities rather than rigid prescriptions for fixed park categories, and Section 5.3 lists the low silhouette coefficient as a study limitation. Across the manuscript, we now treat the K-means results as attribute profiling groups rather than strong latent types. These revisions directly address your concern about overinterpretation under low clustering quality.

4) In the Discussion section, delve deeper into the limitations imposed by the R² of 0.433 and suggest how future research might incorporate more variables to improve explanatory power.

Response: Thank you for asking us to discuss the boundary of the model’s explanatory power more explicitly. In the original manuscript, Section 4.4 briefly noted that approximately 43.3% of the variance was explained, but the implications of this result were not developed sufficiently in the Discussion. In the revised manuscript, Section 5.3 now explicitly interprets R² = 0.433 as meaningful but not exhaustive explanatory power. We also specify several omitted or insufficiently captured sources of variation, including weather, seasonality, special events, waiting time, promotional activities, companion composition, and sociodemographic differences. We then outline directions for future research, especially the integration of time-related, behavioral, and process-management variables. This revision creates a direct link between the model results in Section 4.4 and the limitations discussion in Section 5.3, thereby addressing your request in a more systematic way.

5) Ensure all citations are uniformly formatted and complete (e.g., check if reference [36], a Chinese policy document, meets the journal’s formatting requirements).

Response: Thank you for pointing out both the age profile and the formatting consistency of the references. We addressed both issues. In the original manuscript, many sources were dated before 2020, and the formatting of DOIs, URLs, policy documents, and software/platform references was inconsistent. In the revised manuscript, we added several newer studies in the Introduction, Literature Review, and Discussion, including recent work on tourism geographies, crowding, urban emotion, and spatial analytics. We also standardized the formatting of the references as far as possible, including DOI completion and normalized URL presentation. For policy documents and software/platform sources without DOI information, we adopted a consistent institutional-author–year–title–link format. These revisions were made throughout Sections 1, 2, 5.1, and the reference list, and they also respond to Reviewer #2’s concern that the literature was insufficiently up to date.

6) Figure captions should describe the content more clearly. For instance, the caption for Figure 7 could be revised to "Partial dependence plots illustrating the nonlinear effects of (a) IP theme intensity, (b) green space, and (c) service quality on predicted positive emotion scores."

Response: Thank you for this concrete suggestion. We revised the figure titles/captions and the corresponding in-text explanations to make the relationship between each figure and the related variables or models more explicit. In the original manuscript, the captioning was relatively brief, and much of the reader’s understanding depended on the order of subplots and the surrounding paragraph text. In the revised manuscript, the text in Sections 4.4 and 4.5 now explicitly links Figure 7 to the partial dependence relationships for IP strength, green-space leisure, and service quality, and links Figure 8 to the supplementary spline and segmented regression results. We also revised the wording of the figure titles/captions accordingly. In addition, because Figures 1, 2, 4, and 5 were redrawn using open-source map data and improved for clarity, the final submission package will include the updated figure files with the clearer captions. This revision directly addresses your suggestion regarding caption clarity and figure–text correspondence.

Reviewer #2

Reviewer #2: The study analyzes theme parks in Shanghai, aiming to quantify visitors’ positive emotions and identify associated attributes. To achieve this, it integrates online analytics, POI data, and geospatial information to conduct spatial pattern analysis and develop interpretable predictive models. However, these spatial analyses appear to have been insufficiently explored in the thematic figures presented and in the discussion of their effectiveness.

Therefore, we suggest some revisions to the text and figures presented.

Response: We thank the reviewer for pointing out the overall issue that “the spatial analysis thread is insufficient.” We agree that in the original manuscript, spatial methods appeared more as technical steps rather than being fully developed into a research question and interpretive framework. To address this, the revised manuscript systematically reinforces the Introduction, Methods, Results, and Discussion sections around the core motivation: “Why must we understand emotional differences in theme parks from an urban spatial scale?”

The original manuscri

Attachment Submitted filename: Response to Reviewers.docx

# Peer review: Decision Letter 1

**DOI:** 10.1371/journal.pone.0344051.r003

**Authors:** Renwu Wu

2 Jun 2026

How Do Theme Park Attributes Influence Visitors’ Positive Emotions? Nonlinear Effects and Spatial Heterogeneity in Shanghai’s Theme Parks

PONE-D-26-07392R1

Dear Dr. Gao,

We’re pleased to inform you that your manuscript has been judged scientifically suitable for publication and will be formally accepted for publication once it meets all outstanding technical requirements.

Within one week, you’ll receive an e-mail detailing the required amendments. When these have been addressed, you’ll receive a formal acceptance letter and your manuscript will be scheduled for publication.

An invoice will be generated when your article is formally accepted. Please note, if your institution has a publishing partnership with PLOS and your article meets the relevant criteria, all or part of your publication costs will be covered. Please make sure your user information is up-to-date by logging into Editorial Manager at Editorial Manager® and clicking the ‘Update My Information' link at the top of the page. For questions related to billing, please contact billing support.

If your institution or institutions have a press office, please notify them about your upcoming paper to help maximize its impact. If they’ll be preparing press materials, please inform our press team as soon as possible -- no later than 48 hours after receiving the formal acceptance. Your manuscript will remain under strict press embargo until 2 pm Eastern Time on the date of publication. For more information, please contact onepress@plos.org.

Kind regards,

Renwu Wu

Academic Editor

PLOS One

Reviewers' comments:

Reviewer's Responses to Questions

**Comments to the Author**

1. If the authors have adequately addressed your comments raised in a previous round of review and you feel that this manuscript is now acceptable for publication, you may indicate that here to bypass the “Comments to the Author” section, enter your conflict of interest statement in the “Confidential to Editor” section, and submit your "Accept" recommendation.

Reviewer #2: All comments have been addressed

**********

2. Is the manuscript technically sound, and do the data support the conclusions?

The manuscript must describe a technically sound piece of scientific research with data that supports the conclusions. Experiments must have been conducted rigorously, with appropriate controls, replication, and sample sizes. The conclusions must be drawn appropriately based on the data presented.

Reviewer #2: Yes

**********

3. Has the statistical analysis been performed appropriately and rigorously?

Reviewer #2: Yes

**********

4. Have the authors made all data underlying the findings in their manuscript fully available?

The PLOS Data policy requires authors to make all data underlying the findings described in their manuscript fully available without restriction, with rare exception (please refer to the Data Availability Statement in the manuscript PDF file). The data should be provided as part of the manuscript or its supporting information, or deposited to a public repository. For example, in addition to summary statistics, the data points behind means, medians and variance measures should be available. If there are restrictions on publicly sharing data—e.g. participant privacy or use of data from a third party—those must be specified.

Reviewer #2: Yes

**********

5. Is the manuscript presented in an intelligible fashion and written in standard English?

PLOS ONE does not copyedit accepted manuscripts, so the language in submitted articles must be clear, correct, and unambiguous. Any typographical or grammatical errors should be corrected at revision, so please note any specific errors here.

Reviewer #2: Yes

**********

6. Review Comments to the Author

Please use the space provided to explain your answers to the questions above. You may also include additional comments for the author, including concerns about dual publication, research ethics, or publication ethics. (Please upload your review as an attachment if it exceeds 20,000 characters)

Reviewer #2: I understand that the authors have made the corrections suggested in the first evaluation. And especially the analyses and discussions regarding the spatial approach in relation to the Park areas are now honest regarding the possibilities and many limitations in relation to the variables considered. I believe that the article can be ACCEPTED for publication in the journal.

**********

7. PLOS authors have the option to publish the peer review history of their article (what does this mean?). If published, this will include your full peer review and any attached files.

If you choose “no”, your identity will remain anonymous but your review may still be made public.

**Do you want your identity to be public for this peer review?**   For information about this choice, including consent withdrawal, please see our Privacy Policy.

Reviewer #2: No

**********

Attachment Submitted filename: REVIEWER - R1 - PONE-D-26-07392.pdf

# Editor report: Acceptance letter

**DOI:** 10.1371/journal.pone.0344051.r004

**Authors:** Renwu Wu

PONE-D-26-07392R1

PLOS One

Dear Dr. Gao,

I'm pleased to inform you that your manuscript has been deemed suitable for publication in PLOS One. Congratulations! Your manuscript is now being handed over to our production team.

At this stage, our production department will prepare your paper for publication. This includes ensuring the following:

* All references, tables, and figures are properly cited

* All relevant supporting information is included in the manuscript submission,

* There are no issues that prevent the paper from being properly typeset

You will receive further instructions from the production team, including instructions on how to review your proof when it is ready. Please keep in mind that we are working through a large volume of accepted articles, so please give us a few days to review your paper and let you know the next and final steps.

Lastly, if your institution or institutions have a press office, please let them know about your upcoming paper now to help maximize its impact. If they'll be preparing press materials, please inform our press team within the next 48 hours. Your manuscript will remain under strict press embargo until 2 pm Eastern Time on the date of publication. For more information, please contact onepress@plos.org.

You will receive an invoice from PLOS for your publication fee after your manuscript has reached the completed accept phase. If you receive an email requesting payment before acceptance or for any other service, this may be a phishing scheme. Learn how to identify phishing emails and protect your accounts at https://explore.plos.org/phishing.

If we can help with anything else, please email us at customercare@plos.org.

Thank you for submitting your work to PLOS ONE and supporting open access.

Kind regards,

PLOS ONE Editorial Office Staff

on behalf of

Dr. Renwu Wu

Academic Editor

PLOS One
